# A PKA-selective inhibitor captures an open but more ordered conformation of the PKA catalytic subunit

**DOI:** 10.1073/pnas.2536312123

**Published:** 2026-05-07

**Authors:** Jessica G. H. Bruystens, Jian Wu, Gerald Tan, Daniela Bertinetti, Hans-Michael Zenn, Bastian Zimmermann, Lisa Chen, Johannes Köckenberger, Federica Massaro, Banumathi Sankaran, Matthew S. Walters, Gianluigi Veglia, Fleur M. Ferguson, Friedrich W. Herberg, Susan S. Taylor

**Affiliations:** ^a^Department of Pharmacology, University of California San Diego, La Jolla, CA 92093; ^b^Department of Biochemistry, University of Kassel, Kassel 34109, Germany; ^c^Biaffin GmBH & CoKG, Kassel 34132, Germany; ^d^Department of Chemistry and Biochemistry, University of California San Diego, La Jolla, CA 92093; ^e^ Department of Biochemistry, Molecular Biology, and Biophysics, University of Minnesota; ^f^Molecular Biophysics and Integrated Bioimaging, Berkeley Center for Structural Biology, Lawrence Berkeley National Lab, Berkeley, CA 94720; ^g^Skaggs School of Pharmacy and Pharmaceutical Sciences, University of California San Diego, La Jolla, CA 92093; ^h^Department of Biochemistry and Molecular Biophysics, University of California San Diego, La Jolla, CA 92093

**Keywords:** cAMP-dependent protein kinase, catalytic subunit, PKA-selective inhibitor, crystal structure, SPR measurement

## Abstract

The structure of the catalytic subunit of cAMP-dependent protein kinase (PKA-C), a prototype for the protein kinase superfamily, laid the foundation for the development of targeted kinase inhibitors. Here we describe the structure and biophysical characterization of a PKA-C complex with BLU0588, a small PKA-selective inhibitor. The high-resolution crystal structure not only captures the inhibitor’s unusual T-shaped geometry, but also shows how the four rings of BLU0588 serve as surrogates for ATP’s adenosine and phosphate-organizing sites. Each site contains two subsites. BLU0588’s planar azaindole and pyridine rings, which are buried beneath the glycine-rich loop in a hydrophobic shell at the base of the active site cleft, fill the adenine and ribose subsites. In contrast, BLU0588’s indane and pyrrolidine rings fill the phosphate-organizing site. The indane ring occupies the α/β-phosphate organizing site while the pyrrolidine ring fills the Mg/γ-phosphate organizing site. The structure also shows how BLU0588 nucleates an open but stable conformation of the entire hydrophobic architecture of the N- and C-lobes. In addition to potently blocking phosphoryl transfer activity, BLU0588 also abolishes the synergistic high-affinity binding of the physiological pseudosubstrate inhibitor, protein kinase inhibitor. The residence time of BLU0588, measured by surface plasmon residence, contributes to its picomolar affinity and is distinct from H89, a commonly used but more promiscuous PKA inhibitor. These molecular insights provide a valuable framework for dissecting the organization of the active site cleft as well as different strategies for the rational design of more potent and selective kinase inhibitors in general.

## Significance

A potent protein kinase A (PKA)-selective kinase inhibitor, BLU0588, stabilizes the PKA catalytic subunit in an unusual open conformation and also abolishes the synergistic high-affinity binding of the physiological pseudosubstrate inhibitor peptide, protein kinase inhibitor (PKI). BLU0588 is deeply buried in an extended hydrophobic shell under the kinase-specific glycine-rich loop. Its four hydrophobic rings serve as a surrogate for the entire ATP binding site including the adenosine and phosphate-organizing subsites. Unlike H89, a more promiscuous but commonly used PKA inhibitor that has a rapid on-/off-rate, BLU0588 displays a remarkably slow off-rate and a rapid biphasic on-rate. The molecular features of our BLU0588 bound PKA complex provide therapeutic strategies for designing inhibitors to any protein kinase, not just PKA.

Kinases function by phosphorylating substrates, thereby regulating numerous signaling pathways essential for cellular activity. In protein kinase A (PKA), the catalytic subunit (PKA-C) is held in an inhibited state through association with regulatory (R) subunits. Binding of cyclic adenosine monophosphate (cAMP) to the R-subunits then unleashes the catalytic activity of PKA-C. Additional protein inhibitors include the pseudosubstrate heat stable protein kinase inhibitor (PKI) (1) and, as recently reported, the atypical G-protein-coupled receptor, Smoothened (2). PKA, based on extensive structural and biochemical characterization, has emerged as a prototypical model for the entire kinome (3). Structures of PKA-C were the first to reveal the highly conserved kinase-fold responsible for adenosine triphosphate (ATP) binding and phosphoryl transfer (4, 5). PKA’s kinase core, conserved in all protein kinases, is composed of a smaller N-terminal lobe (N-lobe) and a larger C-terminal lobe (C-lobe) with the ATP-binding site sandwiched in the cleft between both lobes. The adenine ring is buried in a hydrophobic shell at the base of the cleft while the P-site residue is at the outer edge of the cleft. Substrates and substrate inhibitors, like the RII subunits, have a Ser or Thr at the P-site, while pseudosubstrates like PKI and RI subunits have an Ala or Gly at the P-site and bind ATP and the peptide/proteins with synergistic high affinity (6, 7) Key structural motifs within the kinase core include the glycine-rich loop (G-loop), the αC-helix, the DFG (Asp184-Phe185-Gly186) motif within the activation loop and the YRD (Tyr164-Arg165-Asp166) motif that precedes the catalytic loop. Lys72, Glu91, and Asp184 form a regulatory triad that organizes the phosphates of ATP and two Mg^2+^ ions (8). The G-loop and the αC-helix that include K72 in the VAIK motif in β3 strand and E91 in the αC-helix, are located in the N-lobe and interact with the α- and β-phosphates of ATP. In contrast, the catalytic machinery including the DFG and YRD motifs are in the C-lobe; Asp184, which binds to the γ-phosphate and the Mg^2+^ ions, is in the DFG motif. These motifs are essential for positioning and coordinating ATP, along with the two magnesium ions, that contribute to catalysis and opening and closing of the cleft or to the high affinity quenching of pseudosubstrate inhibitors. The structural architecture, ligand–substrate binding synergy, community networks, and the dynamically coordinated movements of the committed, uncommitted, and quenched states work together to orchestrate and finely tune PKA-C activity and its regulation (9–15).

**Fig. 1.**
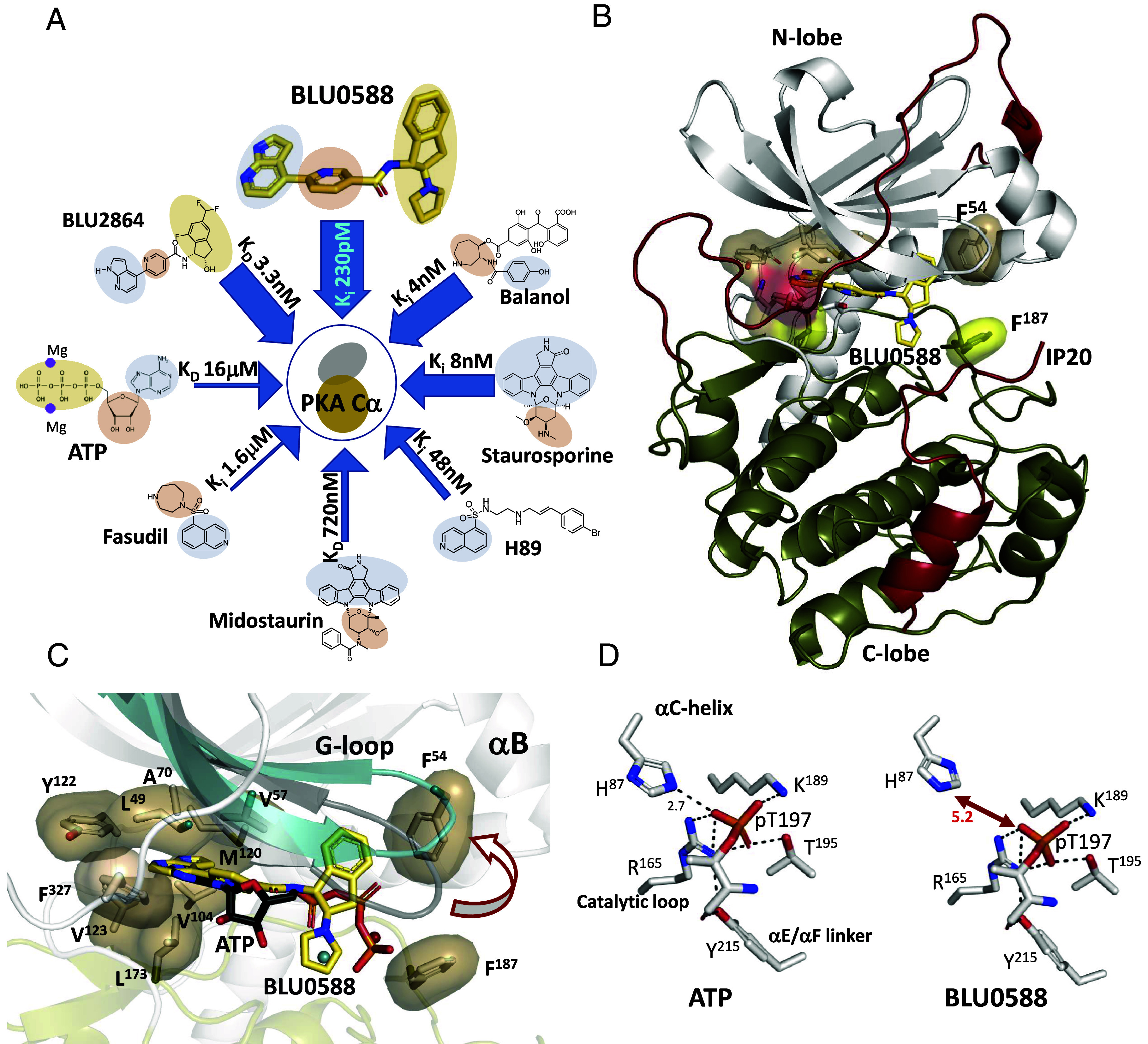
Crystal structure of BLU0588-bound PKA-C. (*A*) Comparison of small molecule PKA-C inhibitors. The surrogate moieties are highlighted: beige for the phosphate-organizing site, tan for the ribose site, and light blue for the adenine site. Binding affinity values were taken from references provided in main text. (*B*) Overall structure of PKA-C with BLU0588 (yellow) bound in the active site and with IP20 (red) docked to the C-lobe. (*C*) Zoom of BLU0588 in the active site overlaid with the ATP-bound C-subunit (PDB ID: 1ATP) highlighting G-loop shifting up. (*D*) Changes of the interaction network nucleated by Thr197 phosphorylation. The increased distances to His87 of the C-helix are highlighted between ATP-bound PKA-C (*Left*) and the BLU0588-bound structure (*Right*).

PKA provided the structural foundation for the field of targeted kinase inhibitors, which has evolved into one of the most prominent areas in cancer therapeutics (16). Most kinase inhibitors target the ATP-binding pocket, exploiting the conserved structural motifs of the kinase core (Fig. 1*A*). To date, over 300 crystal structures of PKA have been deposited in the protein data bank (PDB) and demonstrate the significant malleability of the ATP binding pocket, which can host a variety of chemically and geometrically unique compounds (17). A well-known PKA inhibitor is H89, an isoquinoline high-affinity ATP-competitive inhibitor widely used in cells to block PKA-mediated signaling despite its documented off-target effects (18). Other high-affinity PKA inhibitors include staurosporine and the fungal metabolite balanol, both of which are notoriously promiscuous (Fig. 1*A*) (19–22). Fasudil, another isoquinoline, acts as a dual ROCK/PKA inhibitor but binds PKA with lower affinity (Fig. 1*A*) compared to ROCK. This creates a selectivity window to function in cellular contexts, enabling its use in studies of cardiovascular and neurodegenerative diseases where ROCK is implicated (23). Kinase function and inhibition are also influenced by factors that extend beyond the active site. For example, compounds can differentially modulate PKA-C by altering allosteric substrate and nucleotide-binding cooperativity, highlighting that specificity profiling and binding potency alone cannot fully explain selectivity, inhibitor efficacy, or paradoxical kinase activity in cells (12).

The appreciation of PKA-associated signalopathies has renewed interest in the therapeutic potential for targeting PKA. Down-regulation of PKA-C activity slows the progression of polycystic kidney disease (3, 24, 25) and mutations of the PRKACA gene have been linked to Cushing’s syndrome (26), cardiomyopathies, and more recently, to impaired Hedgehog signaling that leads to Cardioacrofacial Dysplasia (CAFD) (27, 28). Fibrolamellar Hepatocellular Carcinoma (FL-HCC), a rare liver cancer affecting young adults, is driven by a chimeric, but still active, PKA-C that created by a gene fusion with the DnaJ Heat Shock Protein Family Member B1 (*DNAJB1*) (29, 30). To explore the etiology of FL-HCC more deeply, Blueprint Medicines developed a small-molecule ATP-competitive PKA-selective inhibitor, BLU0588 (24, 31). This pharmacokinetically favorable drug successfully targeted tumor cells and PKA-C activity in murine xenograft models and showed the power of selectively targeting PKA in a cellular environment (24). Driven by these findings, we set out to characterize the structure and biophysical properties of BLU0588 interactions with PKA-C. Our 1.55 Å crystal structure of BLU0588 bound to a PKA-C:IP20 complex reveals how BLU0588 forms a network of hydrophobic interactions that stabilizes an open, yet rigid, conformation of the kinase not previously observed. Additionally, we delineate BLU0588’s biophysical binding parameters, define the exceptionally high picomolar affinity and show that BLU0588 abolishes the synergistic high-affinity binding with the pseudosubstrate PKI peptide IP20. Our study describes the molecular features of BLU0588’s binding potency to PKA-C where the four rings of BLU0588 substitute for all four subsites of the ATP-binding site. The hydrophobic adenosine site (AS) includes the adenine/ribose subsites while the phosphate-organizing site also contains two subsites. One subsite organizes the α/β-phosphates while the other organizes the Mg ions and the γ-phosphate. The high-resolution structure of the PKA-C/ BLU0588 complex, together with the biophysical characterization, provides a strategy for developing alternative, small, drug-like inhibitors to target this kinase, or any other kinase.

## Results

### Structure of the C-Subunit in Complex with BLU0588 and IP20.

To elucidate the molecular features driving the inhibition of PKA activity by BLU0588, we solved a crystal structure of a PKA-C:IP20:BLU0588 complex to 1.55 Å resolution (*SI Appendix*, Table S3). The overall conformation of PKA-C displays the canonical bilobal kinase architecture, with the IP20 peptide docked to the C-lobe and clear electron density for BLU0588 in the ATP-binding cleft (Fig. 1*B* and *SI Appendix*, Fig. S1). The BLU0588 scaffold contains two bicyclic rings: a bicyclic azaindole ring fused to a pyridine (nicotinamide) ring and an unusual indane-pyrrolidine bicyclic ring (*SI Appendix*, Fig. S2) (32). The aromatic azaindole ring, extensively used as an adenine mimetic in kinase inhibitors, is anchored in the adenine portion of the ATP binding site while the pyridine ring is anchored to the ribose moiety (Fig. 1*C* and *SI Appendix*, Fig. S3*A*). Together these two rings occupy the extended hydrophobic shell that surrounds the adenosine. The other bicyclic ring lies at the cleft interface that is typically occupied by the phosphates and Mg ions when ATP is present. The indane ring is hydrophobically anchored to the G-loop while the pyrrolidine ring replaces the Mg/phosphate subsite. Although structural predications suggest that a puckered, pseudoequatorial pyrrolidine ring is the lowest energy state in solution (*SI Appendix*, Fig. S1*A*); the electron density of the bound inhibitor clearly shows that the indane and pyrrolidine rings are more planar, with the pyrrolidine adopting a conformation resembling the well-characterized pseudorotation intermediate (*SI Appendix*, Fig. S1*B*) (33). The indane and pyrrolidine rings are oriented perpendicularly to the azaindole-nicotinamide (pyridine) plane, forming a T-shape where the indane ring is wedged under the G-loop at the edge of the active site cleft, while the pyrrolidine ring replaces the space that is usually filled by the P-site, the γ-phosphate and one or two Mg ions (Fig. 1*C* and *SI Appendix*, Fig. S3 *A* and *B*). The molecular geometry of the inhibitor thus hijacks the entire active site of PKA-C: the azaindole and pyridine rings occupy the hydrophobic adenosine pocket, while the indane and pyrrolidine rings occupy the ATP:Mg^2+^ phosphate positioning subsite.

Similar to the adenosine-bound conformation where the cleft is more open and the G-loop is more dynamic (PDB ID: 1BKX) (34), our PKA-C:BL0588 complex displays a partially open active site with the G-loop shifted upward away from the C-lobe (*SI Appendix*, Fig. S3*C*) (34). In contrast, however, the G-loop is very stable. In the closed quenched PKA-C conformation with the pseudosubstrate inhibitor IP20, ATP, and two magnesium ions (PDB ID: 1ATP), the cleft is fully closed with Phe54 in the G-loop forming a hydrophobic shield with Phe187 to sequester the metal coordination site (4). This quenched state captures the synergistic high-affinity binding of the pseudosubstrate and ATP that requires the second inhibitor Mg ion. The indane-pyrrolidine rings of BLU0588 prevent this closure because they are positioned vertically in a pocket otherwise occupied by the horizontally oriented phosphates of ATP (Fig. 1*C* and *SI Appendix*, Fig. S3*A*). Consequently, the Phe54–Phe187 interaction as well as the hydrogen bonding interaction between His87 of the αC-helix and the phosphorylated Thr197 (p-Thr197) in the activation loop, both characteristic features of the closed PKA-C state, are disrupted (Fig. 1*D*).

### BLU0588 Abolishes the Electrostatic Interactions Associated with the Phosphates of ATP.

**Fig. 2.**
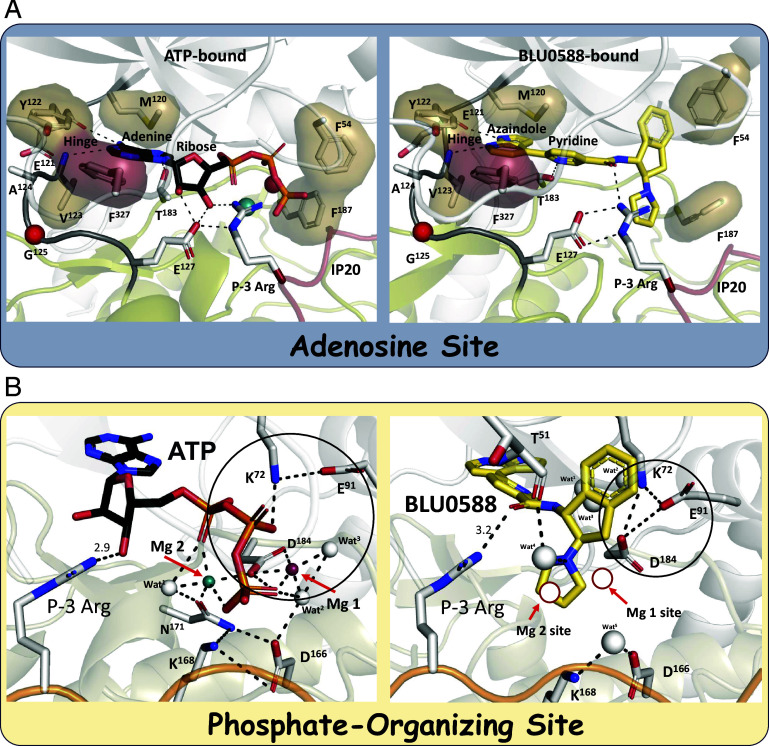
Comparison of the AS and phosphate-organizing site of the BLU0588- vs. ATP-bound PKA-C. (*A*) *Left* panel: AS of the ATP-bound conformation (PDB ID: 1ATP). *Right* panel: The same AS of the BLU0588-bound conformation. The hinge residues (Met120-Glu127) as well as Thr183 are highlighted. All the H-bonds are shown with dashed lines, and the hydrophobic surfaces are shown as shells. The hinge point, G125, is marked as a red ball. (*B*) *Left* panel: Phosphate-organizing site of the ATP-bound conformation, displays the highly coordinated network of Mg^2+^ 1 (magenta), Mg^2+^ 2 (teal), phosphates (red/orange), and waters (gray spheres). The catalytic triad of Asp184, Lys72, and Glu91 is highlighted with a black circle. *Right* panel: The same Phosphate-organizing site of the BLU0588-bound conformation. The space that Mg^2+^ 1 occupies when present is depicted as a white disc.

At the base of the active site cleft, BLU0588 forms hydrogen bonds to the hinge region that links the N- and C-lobes (residues 121 to 126), consistent with PKA-C structures containing the adenine moiety of nucleotides and inhibitors. In this structure, BLU0588’s azaindole is engaged with hydrogen bonds to the main chain hinge residues Glu121 and Val123 (Fig. 2*A* and *SI Appendix*, Fig. S3*B*). BLU0588 also forms a weak hydrogen bond with the side chain of Thr183, which in the ATP-bound structure interacts with the adenine ring (Fig. 2*A* and *SI Appendix*, Fig. S3*B*). In contrast to the ATP-bound structure, however, the extensive electrostatic and hydrogen bonding network that interacts with the rest of the nucleotide is completely missing. To reach a catalytically competent state, PKA-C needs to bind ATP with two Mg^2+^ ions, and in the presence of a pseudosubstrate inhibitor like PKI, it adopts a fully closed conformational state (35). The metal ions coordinate the oxygens on the γ-phosphate of ATP with C-lobe active site residues (Asp184, Asn171, Lys168, and Asp166) and 3 highly ordered water molecules in the predominantly electrophilic charged site (Fig. 2 *B*, *Left*) (35). One Mg^2+^ (Mg-2), referred to as the activating magnesium, is necessary for catalysis while the other (Mg-1) is inhibitory and referred to as the linchpin magnesium (36).

While excess Mg^2+^ was included in the crystallization buffer, there is no density for metals in our BLU0588-bound structure, and this is consistent with many other PKA-C inhibitor-bound structures. The electrostatic interactions that otherwise capture the phosphates of ATP are now spatially replaced by the indane and pyrrolidine rings of BLU0588. Specifically, the Mg-2 site in our structure is occupied by the pyrrolidine ring of BLU0588, which extends down into the γ-phosphate site. The pyrrolidine ring is thus adjacent to the position of the in the active PKA-C structure coordinated linchpin Mg^2+^ (Mg-1), γ-phosphate, and the catalytic triad residues (Lys72, Glu91, and Asp184) (Fig. 2 *B*, *Right* and *SI Appendix*, Fig. S4). Lys72 in β-strand 3 and Glu91 in the C-helix from the N-lobe form a tight salt-bridge cluster directly with Asp184 in the magnesium-positioning loop (DFG motif) of the C-lobe (Fig. 2 *B*, *Right*). Confirmed with the kincore webserver, the DFG and the C-helix salt bridge are considered “in,” categorizing BLU0588 as a Type 1 inhibitor (37). Thus, although the regulatory triad residues are tethered in an electrostatic cluster with each other, they are not interacting with BLU0588 and are instead shielded behind the indane moiety. The interactions of Asp184 with the catalytic machinery in the C-lobe are completely severed by BLU0588. In our BLU0588-bound structure the regulatory triad (black circle in Fig. 2) is no longer part of the catalytic machinery; instead, it is anchored to the α-C helix and stabilizing an open conformation at the cleft interface.

The importance of conserved water molecules in the active site network of PKA-C was previously described (38–40). A network of 5 highly coordinated water molecules occupies the BLU0588-bound PKA-C active site with striking positive Fo-Fc density visible in the omit maps (*SI Appendix*, Fig. S4*A*). Three of the waters are located deep in the active site cleft and are positioned right behind the nicotinamide moiety of the inhibitor (W^1-3^). Another water molecule is interacting with and bridges the pyrrolidine nitrogen of BLU0588 and the backbone oxygen of Thr51 in the G-loop (W^4^, Fig. 2 *B*, *Right* and *SI Appendix*, Fig. S4*B*). The last active-site water molecule (W^5^) forms hydrogen bonds with Lys168 and Asp166 in the C-lobe (Fig. 2 *B*, *Right*). In the ATP-bound state, Lys168 and Asp166 coordinate with Asn171, which is the active-site C-lobe tether that links to the metal-phosphate positioning network (Fig. 2 *B*, *Left*) (35). BLU0588 thus has no hydrogen bonds or electrostatic interactions with the catalytic machinery that otherwise positions (N-lobe/Lys72 and Glu91) and transfers (C-lobe/Asp184) the γ-phosphate from ATP to a protein substrate. Because this coordination is missing, the sensing mechanisms within the active-site that bridge catalysis with opening and closing of the catalytic cleft are uncoupled. In addition to positioning and stabilizing the active site residues, the water molecules likely aid to neutralize the electrophilic charged C-lobe active site surface. Although electrostatic interactions are typically recognized as critical features for high-affinity kinase inhibitors, these interactions are completely missing in the BLU0588:PKA-C complex (41).

### IP20 Is Dynamic and Uncoupled from the Active Site.

**Fig. 3.**
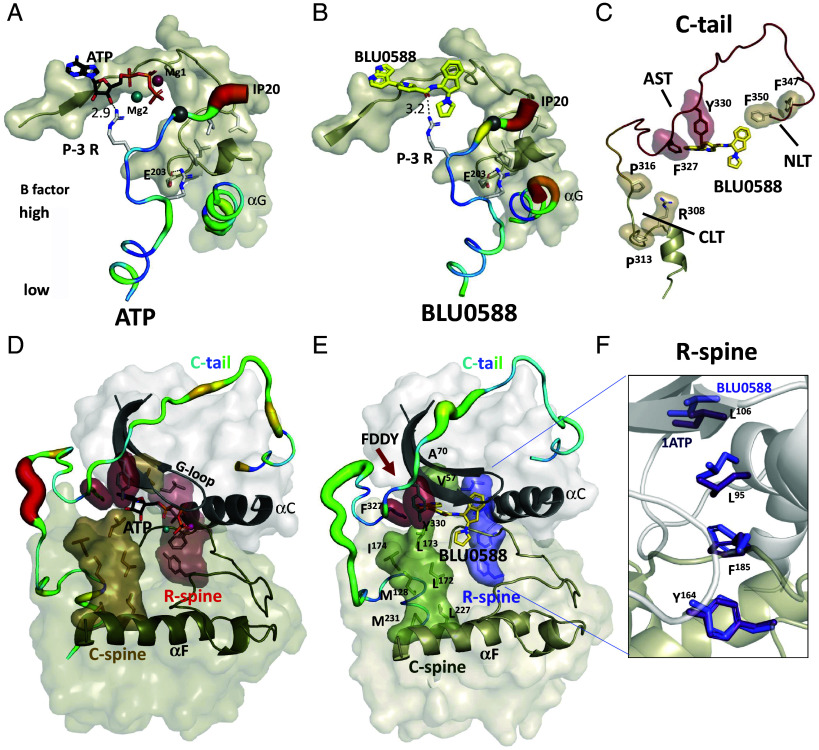
B-factors indicate changes of PKA-C:BLU0588 dynamics. (*A*) The B-factor of IP20 in the ATP-bound conformation. B-factors of the color bar scale range from 20 to 100 for the ATP-bound structure. (*B*) The B-factor of IP20 in the BLU0588-bound conformation. The P-site is more flexible. B-factors of the color bar scale range from 10 to 70 for the BLU0588-bound structure. (*C*) C-tail (red) of PKA-C is ordered in the BLU0588-bound conformation. The hydrophobic tethers (CLT, AST, NLT) are shown. The comparison of the B-factors in the C-tails of ATP-bound (*D*) with BLU0588-bound (*E*) PKA-C structure. The C-spine (tan or green) and R-spine (red or blue) are highlighted. The C-tail in the BLU0588-bound conformation is much more stable. (*F*) Alignment of the R-spine residues in the BLU0588-bound (light violet) to the ATP-bound structure (dark purple).

Although IP20, a peptide derived from PKI (5-24), is bound in our BLU0588:PKA-C structure with side chain densities for Gly14 to Ile22, it is docked less stably and is more dynamic than in the ATP:Mg^2+^ bound state. The pseudosubstrate motif is accommodated within the substrate-binding groove of the C-lobe’s catalytic core, albeit with higher B-factors and density for the His23 side chain and for Asp24 are missing (Fig. 3 *A* and *B*). In contrast to the ATP-bound conformation where the P-site Ala is trapped with a Mg-1-dependent high affinity, the P-1 through P+4 sites of IP20 in the BLU0588 complex are not well-ordered and the side chain of the P+1 Ile22, based on the high B-factors, is no longer stably anchored to the P+1 pocket and to Tyr247 in the G-helix. The B-factors for the N terminus of the G-helix, which is positioned just below the side chain of Ile22 in IP20, are also increased. In addition, the N-terminal helix (Thr5-Ser13) of IP20 is shifted away from the D-helix and closer to the αFαG loop compared to the ATP/Mg^2+^ structure. Yet, there are interactions of IP20 sufficient for low-affinity binding of IP20 in the crystal lattice where 20 times excess peptide to PKA-C is present. While alignment of the peptide’s pseudophosphorylation site with the catalytic loop of PKA-C is maintained, the only contact of IP20 with BLU0588 is the weak hydrogen bond from the P-3 Arg to the aldehyde oxygen of the nicotinamide moiety (Fig. 3*B*). Therefore, the IP20 peptide is essentially excluded from the active site.

### BLU0588 Nucleates an Extended Hydrophobic Network.

Hydrophobic features play a crucial role in the assembly and catalytic function of PKA-C. In the fully closed and active PKA-C structure (PDB ID: 1ATP) (35) the C-tail (residues 300 to 350), which is conserved in all AGC kinases (42), is anchored to the N- and C-lobes of the kinase core with defined clusters of hydrophobic amino acids: the N-lobe Tether (NLT), C-lobe Tether (CLT), and Active Site Tether (AST). In active kinases, the AST is sensitive to nucleotide binding. In the PKA-C:ATP/Mg^2+^:IP20 complex, the AST that contains the essential FDDY motif (residues 327 to 330) is stably anchored to the N-Lobe (Fig. 3*C*). In contrast, in the apo protein (PKA-C without peptide, nucleotide, and metal) the AST is disordered (16). In our PKA-C:BLU0588 structure, while displaying an open conformation, the AST is ordered, and the two residues of the FDDY motif, Phe327 and Tyr330, are positioned as part of the hydrophobic shell that now surrounds the azaindole ring of BLU0588 in place of the adenine ring of ATP (Fig. 3 *C* and *E*) and the entire C-tail shows a reduction in B-factor dynamics (Fig. 3*E*).

The R-spine (regulatory spine) and C-spine (catalytic spine) are additional important and conserved integral hydrophobic features of every kinase (43–45). In the presence of ATP, the R- and C-spine span both lobes to form contiguous hydrophobic scaffolds that connect most of the motifs known to be important for catalytic function (Fig. 3*D*) (11). In our BLU0588 bound structure, the C-spine’s hydrophobic scaffold engulfs and positions the azaindole and pyridine of the inhibitor (Fig. 3*E*). The aromatic rings are thus sandwiched between two highly conserved residues on the top from the N-lobe (Ala70 in β3 and Val57 in β2) and on the bottom from the C-lobe (Leu173) (Fig. 3*E*).

While C-spine alignment is preserved in the BLU0588-bound state, the R-spine shows some small differences (Fig. 3*F*). The R-spine includes four vertically stacked residues, Leu106 in β5 and Leu95 in the C-helix come from the N-lobe while Phe185 in the DFG motif (Mg positioning loop) and Tyr164 in the catalytic loop YRD motif, come from the C-lobe (Fig. 3*D*). Phe185 sits underneath the C-helix as in the closed ATP structure but there is a small shift in the angle of the aromatic ring plane (Fig. 3 *D* and *F*). Consistent with a slightly more open PKA-C conformation, both Leu106 and Leu95 have moved up slightly creating a small gap between the two lobes, but overall, the hydrophobic R-spine is assembled. Furthermore, assembly of the R-spine leads to the convergence of the regulatory triad and while it is not interacting with BLU0588 (Fig. 2 *B*, *Right*) it has formed a stable electrostatic cluster that in turn also supports stabilizing the R-spine.

**Fig. 4.**
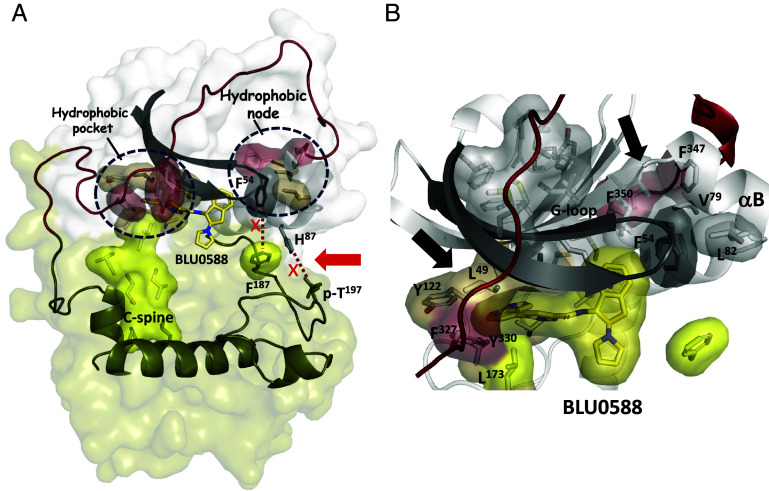
BLU0588 nucleates an extensive hydrophobic network of interactions. (*A*) The hydrophobic residues from the N-lobe (tan), the C-lobe (yellow), and the C-tail (red) surround BLU0588. The C-spine is shown in yellow. In the BLU0588 complex, the hydrophobic shell between Phe54 and Phe187, as well as the H-bond between H87 and pT197, that are present in the ATP-bound conformation, are now broken (marked by red arrow). (*B*) The hydrophobic surface of the N-lobe (gray) when bound with BLU0588. The hydrophobic residues, Phe54 from the G-loop, Val79 and Leu82 from the B-helix, as well as several residues from the C-tail (red) including Phe327, Tyr330, Phe347, and Phe350 (marked by black arrows) are highlighted.

The hydrophobic surface area of the BLU0588 compound is thus buried and connected to the hydrophobic core residues of both the N-lobe and the C-lobe, utilizing in part the spine assemblies and the FDDY tethering amino acids (Phe327 and Tyr330). In addition, Tyr122 and Val123 from the hinge, Val104 in β4, Leu49 in β1 and Met120 (the gatekeeper residue) and Met118 in β5 extend the hydrophobic network around BLU0588 (Fig. 4). This hydrophobic network is further complemented by the indane ring of BLU0588 stacking up against the G-loop (Phe54), which in turn is hydrophobically packed to the B-helix (Val79 & Leu82) (Fig. 4). BLU0588 is thus tightly anchored to the β-sheet and the B-helix in the N-lobe (Fig. 4).

### Binding of BLU0588 Stabilizes the Conformation of PKA-C and Abolishes the Synergistic High-Affinity Binding of IP20.

**Fig. 5.**
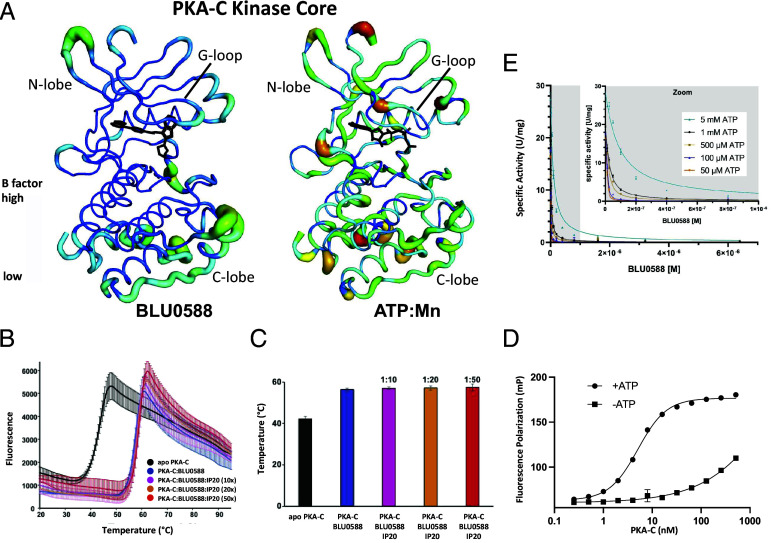
Biochemical analysis of BLU0588 binding to PKA-C. (*A*) B-factor comparison of the kinase core between BLU0588-bound (*Left*) and ATP-bound (*Right*) PKA-C. B-factors of the color bar scale range from 20 to 100 for the ATP-bound structure and from 10 to 70 for the BLU0588-bound structure. (*B*) Thermal denaturation profile of PKA-C in the absence (black) and in the presence of BLU0588 (blue). Three different ratios of PKA-C:IP20 [1:10 (pink), 1:20 (orange), and 1:50 (red)] were tested. (*C*) Histogram representing the Tm (°C) values of PKA-C apo (42 ± 1), with 50 µM BLU0588 (56 ± 1), and at three different molar ratios of PKA-C:IP20, 1:10 (57 ± 1), 1:20 (57 ± 1), and 1:50 (58 ± 2). (*D*) Determination of the apparent K_i_ for BLU0588. Phosphorylation of the peptide substrate Kemptide was inhibited by adding BLU0588 with various ATP concentrations as indicated in the legend. Enzyme activities with inhibitor concentrations below 1 µM are enlarged in the inlet (gray background). The apparent K_i_ was calculated using global fit of the Morrison equation for tight binding inhibitors (see methods). Data fitted globally for 50 µM, 100 µM, 500 µM, and 1 mM ATP yielded an R^2^ > 0.993; including 5 mM ATP lowered the R^2^-value to R^2^ < 0.981, suggesting additional effects at high ATP not explained by a simple competition with ATP/Mg^2+^. (*E*) FP assay to analyze the binding of fluorescein-labeled IP20 peptide to PKA-C (0.25 to 512 nM) in buffer containing 10 mM MgCl_2_ in the presence (●) or absence (■) of 1 mM ATP. Each experiment was tested in triplicate and the data were analyzed with GraphPad Prism9.

A comparison of the B-factors between the ATP-bound and BLU0588-bound structures reveals a striking and significant increase in the rigidity of PKA-C upon binding BLU0588. Not only is the C-tail more stable (Fig. 3*E*) but the motion of the entire kinase core is restricted (Fig. 5*A* and *SI Appendix*, Fig. S5), suggesting that this inhibitor may have global stabilizing effects on the enzyme relative to the ATP-bound state. To confirm this hypothesis, we performed thermal shift assays on PKA-C in the absence and presence of BLU0588 at 1:10 molar ratio. Consistent with our structural data, the thermal stability assays show that BLU0588 stabilizes the overall kinase fold significantly more than the nucleotide. By fitting the melting curves using the Boltzmann equation, we determined that BLU0588 changes the melting temperature (T_m_) of the enzyme from 42.7 ± 0.9 °C to 57.0 ± 0.7 °C (ΔT_m_ ~ 14 °C) (Fig. 5*B*). In contrast, the binding of IP20 in the absence of nucleotide enhances the thermostability of the enzyme by only ~3 °C (*SI Appendix*, Fig. S6*A* and Table S1). Likewise, upon saturating PKA-C with the ATP analog AMP-PNP, we observed an enhancement of thermostability by only ~2 °C (T_m_ = 44.6 ± 0.8 °C) (*SI Appendix*, Fig. S6*B*) These findings indicate that, upon binding BLU0588, PKA-C adopts a thermodynamically more stable conformation than the AMP-PNP bound state.

Moreover, our structural data suggest that the inhibitory peptide IP20 remains quite dynamic within the substrate binding pocket, and that this pseudosubstrate may not contribute to the overall stability of PKA-C. To address this, we measured the thermal denaturation profiles of the PKA-C/BLU0588 complex in the presence of increasing concentrations of IP20 and found no appreciable change in the T_m_ value, confirming that peptide binding does not contribute to the overall stability of the enzyme (Fig. 5*C*).

A hallmark of PKA signaling is the allosteric coupling between the ATP and substrate binding sites (14). Specifically, nucleotide binding to the kinase in the presence of a second Mg^2+^ ion enhances the affinity for substrates, PKI, and RIα (43, 44, 46). The lack of stabilization of the PKA-C/BLU0588 complex upon PKI binding suggests that the synergy between BLU0588 and PKI may be disrupted. To test this, we performed isothermal titration calorimetry (ITC) measurements to determine the dissociation constants (*K*_d_) of IP20 to PKA-C with and without BLU0588 (*SI Appendix*, Fig. S7 and Table S2). For the PKI binding to the apo kinase, we found an average *K*_d_ of 3.8 µM, while for the PKA-C saturated with BLU0588 we determined an average *K*_d_ of 2.9 µM. From these values we obtained a cooperativity coefficient (σ=KdapoKdBLU0588) of ~1.3, indicating the absence of binding synergy between BLU0588 and the PKI peptide. A fluorescence polarization (FP) assay also shows the loss of the synergistic binding of fluorescently labeled IP20 (FAM-IP20) that is seen in the presence of ATP/Mg^2+^ (Fig. 5*D* and *SI Appendix*, Fig. S8*A*). IP20 does not bind PKA-C in the presence of BLU0588 in these assay conditions, and furthermore, addition of BLU0588 outcompetes 50 µM ATP and prevents binding of FAM-IP20 to the C-subunit (*SI Appendix*, Fig. S8*B*).

To further elucidate the functional consequences of BLU0588-binding the inhibitory potency was evaluated via a spectrophotometric kinase assay using Kemptide as a substrate (47) in the presence of 10 mM MgCl_2_ and at increasing ATP concentrations. Under our standard assay conditions, the PKA-C concentration exceeds the determined IC_50_ literature value for BLU0588, which means that with a classic sigmoidal dose–response analysis the calculated IC_50_ value is error-prone (48, 49). For this reason, we applied a global fit analysis to the inhibition data at different ATP concentrations using the Morrison Equation (50) (see Methods). This equation accounts for the characterization of tight-binding enzyme inhibition because it does not assume that the free concentration of the inhibitor equals the total inhibitor concentration. The resulting apparent inhibitory constant (K_i_) of BLU0588 was determined to be 230 ± 10 pM (Fig. 5*E*) supporting the data by Schalm et al. (24). Fitting the experimental data for BLU0588 inhibition at various ATP concentrations (50 µM, 100 µM, 500 µM, and 1 mM) yielded a fit with high accuracy (R^2^ > 0.993). Notably, BLU0588 was able to almost completely inhibit the activity of 20 nM PKA-C even in the presence of a 1,000 to 5,000 fold molar excess of ATP/Mg^2+^.

**Fig. 6.**
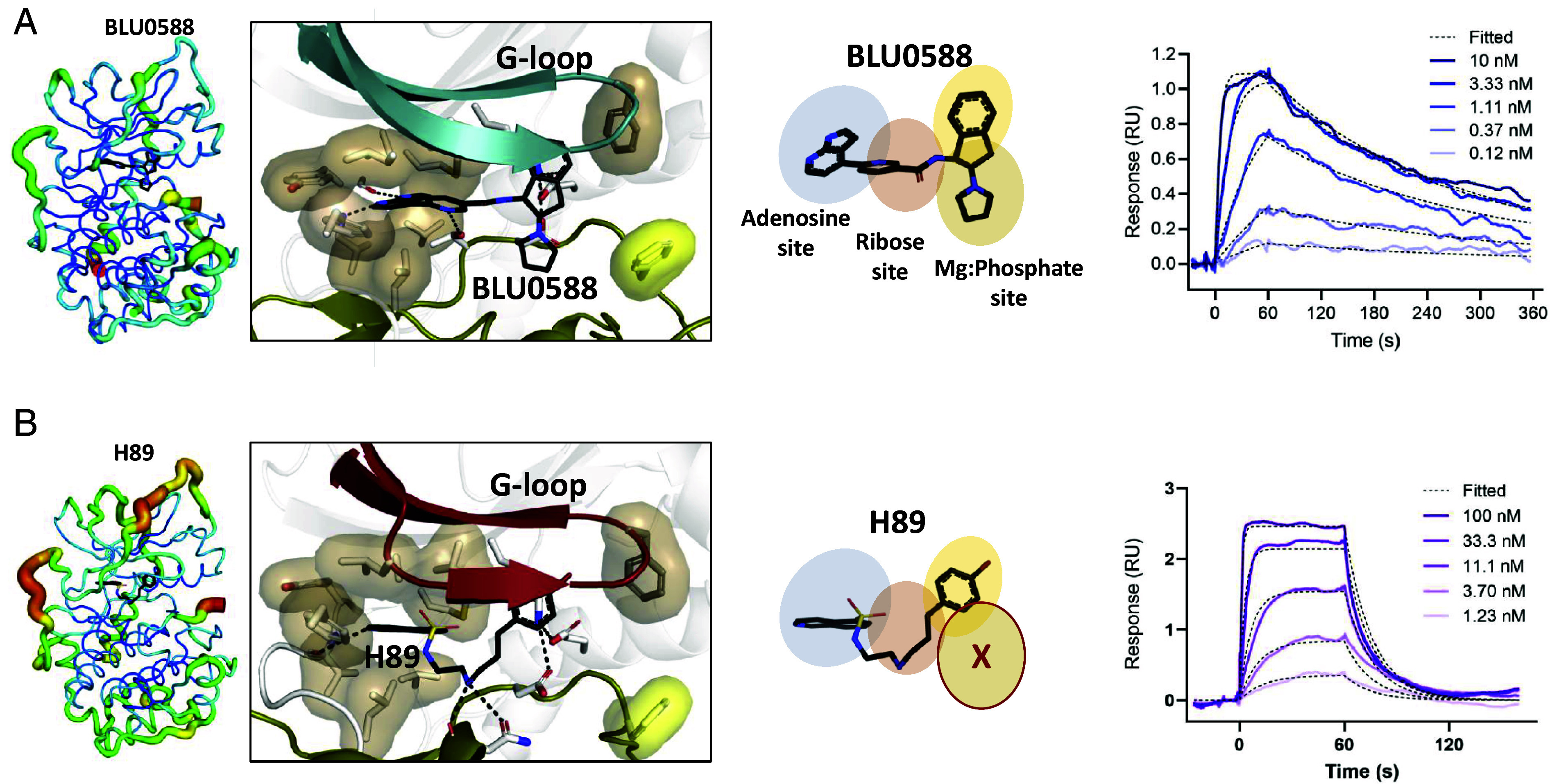
The conformational, dynamic, and kinetic differences between BLU0588 (*A*) and H89 (*B*). *Left* panels: Comparison of the PKA-C B-factor dynamics when bound to the respective inhibitor. *Middle* panels: Inhibitor-bound active site interactions and occupation of each PKA-C subsite. *Right* panels: SPR sensorgrams show the inhibitor’s association and dissociation for BLU0588 and for H89 using a concentration series of the respective inhibitors interacting with captured FSS-tagged PKA-C. Dashed lines are fits to determine the respective rate constants (k_ass_ and k_diss_) and the experimental R_max_ for both inhibitors. Representative experiment of n = 2 experimental setups.

To characterize in more detail the direct binding properties of BLU0588, we performed an SPR-based assay with PKA-C captured onto a sensor chip via an FSS-tag (human PKA-Cα N-terminally tagged with a FLAG- and two Streptags) followed by injection of BLU0588 over the sensor surface. The sensorgram illustrates the very fast association between BLU0588 and immobilized PKA-C (Fig. 6 *A*, *Right*). Because the interactions were strongly influenced by mass transfer limitation (51), surface densities of PKA-C were extremely reduced to a maximum binding signal of 1 RU for BLU 0588 (Fig. 6 *A*, *Right*). These extremely fast on rates could still be accurately determined with a Biacore^TM^ 1S+ instrument (Cytiva). Data were fitted employing a 1:1 Langmuir binding model yielding a k_ass_ of 26 × 10^6^ M^−1^s^−1^ ± 1.8 × 10^6^ M^−1^s^−1^ and a k_diss_ of 5.6 × 10^−3^ s^−1^ ± 0.7 × 10^−3^ s^−1^ yielding a K_D_-value of 0.21 nM ± 0.01 nM. Stoichiometries of the binding partners were calculated based on their respective molecular weights and maximum SPR signal (R_max_). The experimental R_max_ of 1.1 RU for BLU0588 was close to the theoretical R_max_ of 0.9 RU (for calculation see Methods section), suggesting that FSS-PKA-C molecules coupled to the sensor chip forms a 1:1 complex with BLU0588 (52).

Taken together, our data indicate that binding BLU0588 to PKA-C results not only in potent inhibition of substrate phosphorylation but also increases the enzyme’s thermostability and eludes the regulation of PKA-C by PKI.

### Unique Features of the BLU0588:PKA-C Interactions Compared to H89.

Finally, we compared the inhibitory features of BLU0588 with H89, which also binds PKA-C independent of Mg^2+^ ions. H89 is a well-known signaling modulator and it is widely used in cell biology to inhibit PKA-C (53). While H89 and BLU0588 both occupy the same hydrophobic shell that surrounds the adenine and ribose-rings of ATP, H89, unlike BLU0588, is firmly anchored to the catalytic loop through its interactions with Asn171 (H89; PDB ID: 1YDT) (Fig. 6) (54). This prevents the more extensive stabilizing effects of BLU0588(Fig. 6 *A* and *B*, *Left*). More specifically, H89 does not occupy the phosphate-magnesium organizing site (Fig. 6 *B*, *Middle*) and forms only a single H-bond with the kinase’s hinge with its isoquinoline group. The inhibitor’s polar Sulfur-Oxygen-Nitrogen cluster is located within the hydrophobic adenosine binding pocket, whereas the bromocinnamyl moiety of H89 extends out of the adenosine pocket, occupying a completely different chemical space relative to BLU0588. The latter is due to the longer and more flexible scaffold that reaches above the catalytic triad and positions underneath the G-loop engaging in several electrophilic interactions (Fig. 6 *B*, *Middle*). The flexibility of H89 is also due to the rotational degrees of freedom of the diaminoethyl linker that likely contributes to the mobility within the active site of PKA-C. Despite H89 forming several electrostatic interactions with both the N- and C-lobes, it does not stabilize the kinase as effectively as BLU0588. In fact, our thermal shift assays show that 89 binding increases the T_m_ by ~11 °C while BLU0588 increases the T_m_ by ~14 °C (*SI Appendix*, Fig. S9).

Finally, we analyzed the kinetics of association and dissociation of H89 from FSS-tagged PKA-C using surface plasmon resonance (SPR). Using a slightly higher surface density, a k_ass_ of 8.3 × 10^6^ M^−1^s^−1^ ± 0.3 × 10^6^ M^−1^s^−1^ and a k_diss_ of 60 × 10^−3^ s^−1^ ± 6.4 × 10^−3^ s^−1^ yielding a K_D_-value of 7.3 nM ± 1.1 nM was measured (Fig. 6 *B*, *Right*). This indicates an 11-fold faster off rate for H89 compared to BLU0588. Accompanied with a 3.2 slower on-rate, the affinity measured in a direct binding assay of BLU0588 is 35-fold higher compared to H89.

In summary, our data show that H89 binds PKA-C with a significantly lower affinity and does not stabilize the inhibited state as effectively as BLU0588. The latter is due to the different interactions the two inhibitors establish within the kinase core.

## Discussion

**Fig. 7.**
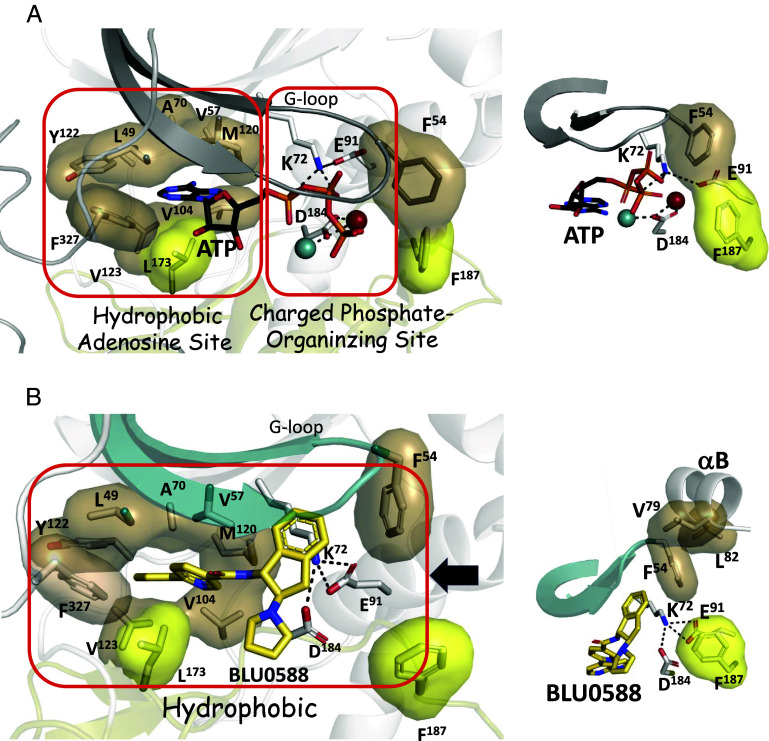
Hydrophobically dominated BLU0588 active site compared to the split electrostatic/hydrophobic active site of ATP-bound PKA-C. (*A*) The active site of the ATP-bound conformation is split into the hydrophobic AS and the electrostatically charged phosphate-organizing site (PS) (*Left*). (*B*) Both the AS and PS sites are hydrophobic with BLU0588 bound to PKA-C. Zoom on the catalytic triad (Asp184, Lys72, Glu91) in both complexes are shown on the *Right* panels. The hydrophobic residues from N-lobe (tan) and C-lobe (yellow) are shown as shells, both G-loop (gray or cyan) are also highlighted. The black arrow shows the disengagement of Phe54 from Phe187 when bound to BLU0588.

The synergistic binding of ATP with the pseudosubstrate inhibitor IP20 and two Mg^2+^ ions, first captured in a crystal structure in 1993, revealed what was at the time a novel nucleotide binding site (11, 55–58). The accepted ATP binding motif in 1993 was the Rossmann fold (also called the P-Loop) with the defining feature being a glycine-rich phosphate binding loop as well as a subsequent lysine that traps the γ-phosphate. Although protein kinases also have a glycine-rich loop and a conserved lysine, the mechanism for trapping the nucleotide is distinct. Protein kinases trap ATP’s adenosine ring in a hydrophobic pocket at the base of the cleft while the γ-phosphate is captured at the edge of the cleft by a network of hydrogen bonds and electrostatic interactions that position the γ-phosphate for transfer to heterologous substrates. In IP20, which is a pseudosubstrate, there is no hydroxyl moiety to accept the γ-phosphate; instead there is synergistic high-affinity binding of ATP and IP20 that is dependent on the inhibitory Mg^2+^ ion (Mg-1) (36, 58). Binding of ATP is thus governed by two sites that are chemically and mechanistically very different (Fig. 7*A*). The hydrophobic site at the base of the cleft, formed mostly by N-lobe residues, binds the adenosine ring and conveys affinity, while the hydrophilic and highly charged phosphate organizing (PO) site controls the positioning of the α- and β-phosphates [K72 (β3) and E91 (αC) in the N-lobe] and the transfer of the γ-phosphate (D184 and the Mg ions in the C-lobe). Opening and closing of the cleft thus serves as the highly regulated switch mechanism that controls kinase activation as well as the high affinity binding of pseudosubstrates (Fig. 7*A*). The protein kinase G-loop is thus distinct from the classic P-loop.

BLU0588 has not only broken PKA-C’s finely tuned machinery that positions the nucleotide (N-lobe) and transfers (C-lobe) the γ-phosphate from ATP to a protein substrate, the indane and pyrrolidine rings of BLU0588 have clearly also defined the two subsites in the PO-site. Instead of the electrophilic phosphate and magnesium-mediated interactions in the PO-site which are essential for catalysis and synergistic high-affinity binding of pseudosubstrates (Fig. 2 *B*, *Right* and Fig. 7*B*), the BLU0588-bound conformation is stabilized primarily by hydrophobic interactions (Fig. 7*B*). Each of the ATP subsites are captured by BLU0588 with each ring optimally complementing the active site cleft. The indane-pyrrolidine rings serve as a novel hydrophobic surrogate for the G-loop and the two Mg^2+^ ions that organize the phosphates of ATP, whereas the azaindole and pyridine rings serve as a surrogate for the adenine and ribose rings. The G-loop is now in an open but ordered conformation while the conserved Asp184 is disengaged from the catalytic machinery (Fig. 7*B*). The side chain of Phe54 at the tip of the G-Loop, serves as a hydrophobic node that connects the tip of the G-Loop to the hydrophobic surface of the B-helix in the N-lobe (Fig. 4). In the absence of the inhibitory Mg^2+^ ion (Mg-1), the G-loop typically becomes flexible but when BLU0588 binds, the indane ring pushes the G-loop up and links it to all of the extended hydrophobic motifs that flank both sides of the β-sheet in the N-lobe including the αB and αC helices (36). The increased B-factors of IP20 in our structure, the lack of binding in our FP-assay, and the thermostability are also consistent with the P-site being uncoupled at the active site cleft.

BLU0588 employs a highly effective molecular strategy with shape complementation allowing for the alignment of hydrophobic elements that fill the entire active site cleft, which includes not only the hydrophobic shell that surrounds the adenosine ring but also the entire PO-site. BLU0588 binding to PKA-C translates to striking biochemical readouts. We observed an increase in thermal stability in the presence of BLU0588, consistent with the low temperature factors, as well as a picomolar K_i_ and tight binding parameters with a fast association and a slow dissociation rate. Although high-resolution SPR measurements unveil that the association of BLU0588 is still limited by mass transfer, rates of association and dissociation at those extreme low surface densities are well within the range of the employed Biacore instrument. This could suggest that BLU0588 must first tether into the active site of PKA-C rather than slipping in like ATP or H89. Furthermore, longer residence times of drugs with their targets, as a result of slower off-rates, is a useful mechanism to extend in-vivo efficacy (59–61). Additionally, a slow-off rate can provide a better therapeutic window if the rates differ relative to off-targets. We predict that BLU0588 also hinders binding of the pseudosubstrate inhibitor RIα. Our analysis indicates that presence of BLU0588 results not only in potent inhibition of PKA-C phosphorylation and an increase in structural rigidity and thermostability, but also in loss of regulation by PKA-C regulatory proteins such as PKI and potentially the RI-subunits.

This contrasts with typical ATP-competitive kinase inhibitors that ineffectively exploit most of the active site pockets. H89, for example, has a much faster off-rate based on SPR, does not induce an open conformation, and does not occupy the full phosphate-organizing site, specifically, the Mg/γ-phosphate/D184 subsite (Fig. 6*B*). For many decades, H89 has been used routinely in cellular assays for inhibition of PKA, even though it is quite promiscuous. There are other signaling pathways that are up- or down-stream of PKA that H89 will inhibit. Our goal here is not to explain the selectivity for BLU0588 binding to PKA nor to design more selective inhibitors; instead, we show BLU0588 can serve as a tool for others that can tease apart these features of the active site cleft. Schalm et al. and Wang et al. already demonstrated the potential of this strategy for PKA-driven diseases such as FL-HCC and PKD (24, 25). Others now need to revisit the cellular H89 studies to confirm which effects are unambiguous due to inhibition of PKA.

We propose that the unique complementarity of the four rings of BLU0588 to the conserved features of the active site cleft accounts for its high potency and unique binding properties. It will now be essential to carry out additional kinetic experiments to rigorously compare BLU0588 to other PKA-C inhibitors. Experiments to define and compare the effect on PKA-C dynamics and substrate binding synergy with other inhibitors using techniques such as NMR, MD simulations, SPR and crystallography are already under way. Taken together, the structural features of BLU0588 elucidated here provide a framework for designing kinase inhibitors with improved cellular efficacy and a valuable tool for the research community revisit and tease apart conserved features of the active site cleft in all protein kinases.

## Experimental Section

### Protein Expression and Purification of PKA-C for Crystallization, FP Assays, and Differential Scanning Fluorimetry (DSF).

Murine or human full-length wild type C-subunit alpha was expressed and purified as previously described (62). PKA-C wild type in a pET-His6-SUMO vector was transformed into Rosetta (DE3) pLysS cells from MilliporeSigma. All subsequent cultures contained 0.1 mg/mL Ampicillin. 3 mL starter cultures were grown from single colonies and used to grow overnight cultures in LB. 1 L liquid LB flasks were then inoculated with overnight culture and grown to an OD_600_ of 0.6 to 1 at 37 °C with shaking at 200 rpm. The temperature was reduced to 16 °C and induced with 0.5 mM IPTG. After ~16 h of expression at 16 °C and 150 rpm shaking cells were harvested and stored at −20 °C.

Cell pellets were resuspended in buffer made of 20 mM Tris pH 8, 300 mM NaCl, 5 mM β-mercaptoethanol (BME) and cOmplete EDTA-free protease inhibitor cocktail tablets from Sigma-Aldrich and lysed with a microfluidizer. The total cell lysate was centrifuged for 1 h at 13,000 rmp and the resulting supernatant was applied to 5 to 20 mL of equilibrated Ni-NTA Agarose (Qiagen) resin. Following incubation at 4 °C overnight, 5 to 8 column volumes of 20 mM Tris pH 8, 300 mM NaCl, and 2 mM BME were applied to wash the resin and then 20 to 50 mL of elution buffer (wash buffer plus 150 mM imidazole) was used to elute the C-subunit. The elution was then incubated with 1:200 molar ratio of previously purified His-tagged Ulp1 to protein for 1 h at room temperature and then dialyzed overnight in 4 L of wash buffer to remove imidazole. The next day the dialyzed protein solution was incubated with 5 to 20 mL of Ni Sepharose resin for 1 h at room temperature to bind the cleaved His-Sumo tag and uncleaved protein. The flow through was collected and concentrated before loading onto a S200 gel filtration column equilibrated with 20 mM MES ph6.5, 200 mM NaCl, and 5 mM DTT. Peak fractions were analyzed by SDS gel electrophoresis and pooled. To improve purity, the protein obtained from the S200 was further purified with a Mono S column. For this, the S200 peak fractions were pooled and dialyzed into Mono S buffer A (20 mM KH_2_PO_4_ pH 6.5 and 5 mM DTT) overnight before loading onto a Uno S6 cation-exchange column (BioRad). The C-subunit was eluted with a gradient of 0 to 500 mM KCl in buffer A and resulted in distinct peaks corresponding to the phosphorylation profile of the C-subunit.

### Expression and Purification of PKA-C for K_i_ Determination and SPR Studies.

For SPR and apparent K_i_ determination an N-terminal FSS-tagged human PKA-Cα was used, where the FSS-tag consists of a FLAG moiety (DYKDDDDK) followed by a double Strep-Tag II (2× WSHPQFEK) as described in ref. 63. FSS-PKA-C was overexpressed in *Escherichia coli* BL21(DE3) cells after induction with 0.4 mM IPTG for 16 h at 20 °C using the expression vector pET30a-FSS-hCa and then purified by affinity chromatography using an IP20-resin as described earlier (45, 46). Purified FSS-PKA-C was then stored at 4 °C in elution buffer (50 mM Tris, pH 7.4, 50 mM NaCl, 200 mM L-arginine, 1 mM EDTA), and the buffer was changed to 20 mM MOPS, pH 7.0, 150 mM NaCl, 2 mM β- mercaptoethanol by dialysis preceding further use.

### Crystallization, Structure Determination, and Refinement.

Peak 3 of the MonoS purified C-subunit, which corresponds to the dual phosphorylation state (S338&T197), was dialyzed overnight into a buffer containing 50mM N,N-bis(2-hydroxyethyl)glycine (BICINE) pH8, 150 mM ammonium acetate, and 10 mM DTT. The protein was then concentrated to 9 mg/mL and used for setting up hanging drop vapor diffusion plates at 4 °C, varying the 2-methyl-2,4-pentanediol (MPD) concentration from 8 to 20% with 0.1 M HEPES pH7 and methanol concentration ranging from 10 to 13%. The hanging drop was composed of 9 mg/mL C-subunit, BLU0588, MgCl_2_, and IP20 with a ratio of 1:5:10:10 respectively. The crystal that gave the best resolution grew with 10% MPD and 10% Methanol. The crystal was harvested, soaked in 20% MPD in mother liquor cryoprotection solution and flash frozen in liquid nitrogen for data collection at the Advanced Light Source, Lawrence Berkeley National Labs (Berkeley, CA) beamline 8.2.2.

Data were processed and scaled with HKL2000 (47) and resulted in space group P 21 21 21 with cell dimensions of a = 69.5 Å, b = 73.1 Å and c = 76.5 Å (*SI Appendix*, Table S1) and a resolution of 1.55 Å. The initial structure model was solved with ccp4 using Phaser (48) for molecular replacement with PDB ID: 1ATP (29) as the search model, omitting ATP:magnesium from the PDB file. The BLU0588 inhibitor was built into the model using Coot’s ligand builder AceDRG (49), followed with real space refinement and ligand restraints file (cif) from Phenix Ready Set (50). Iterative refinement cycles and automatic and manual placement of water molecules was carried out using Coot and Phenix and resulted in a R-work of 0.18 and R-free of .21 (*SI Appendix*, Table S1). All structure figures were made with PyMOL (Molecular Graphics System, Schrödinger LLC).

### DSF.

The thermal denaturation assays were performed using differential scanning fluorimetry. The concentration of wild-type human PKA-C (hPKA-C) was held constant at 5 μM (54) in 20 mM phosphate buffer (pH 6.5) with 90 mM KCl, 10 mM MgCl_2_, 10 mM DTT, and 1 mM NaN_3_. As a reference, we performed thermal denaturation assays on apo hPKA-C and we compared the melting temperature (Tm) with the enzyme in the presence of 50 μM of BLU0588, and increasing concentration of IP20, ranging from 50 to 250 μM. SYPRO Orange dye (Bio-Rad) was added to each sample to a final concentration of 20× (55). For each assay, the total reaction volume was 50 μL. The DSF measurements were carried out in a range of temperatures from 20 to 95 °C using a qPCR instrument (Eppendorf). The melting temperatures were calculated by fitting the denaturation curves using the Boltzmann equation (54).

### ITC Measurements.

All ITC measurements were performed using a TA NanoITC at 27 °C. All samples were prepared using a 20 mM MOPS, 90 mM KCl, 10 mM DTT, 10 mM MgCl_2_, 1 mM NaN_3_ at pH 6.5. Each titration was carried out with an initial sample volume of 350 mL and a concentration of ~100 μM of PKA-C. The concentration of PKI(5-24) in the titrant syringe was 1 mM. For the experiments in the presence of BLU0588, a 1:4 molar ratio of protein:inhibitor was used. All experiments were repeated three times, and the errors were calculated as the SD from the average value of the calculated *K*_d_. The heat of dilution of PKI(5-24) was determined by performing a titration of the peptide into buffer and subtracted to the exotherms. Binding was assumed to be 1:1 and was analyzed using the Wiseman isotherm (64):

$$\frac{d[MX]}{d[X_{\mathrm{tot}}]}=\Delta H^{\circ }V_{0}\left[\frac{1}{2}+\frac{1-(1+r)/2-R_{m}/2}{{\left(R_{m}^{2}-2R_{m}\left(1-r\right)+{\left(1+r\right)}^{2}\right)}^{1/2}}\right],$$

where *d*[*MX*] is the change of the total complex relative to the change of the ligand concentration (*d*[*X*_*tot*_]), *r* is the ratio of the *K_d_* with respect to the total protein concentration, and *R_m_* is the ratio of the total ligand and total protein concentrations. The free energy and entropic contribution to ligand binding were calculated with the following formulas:

$$\Delta G=RT\;\text{ln}\;K_{d},$$

and

TΔS=ΔH-ΔG,

where R is the gas constant and T is the temperature. The cooperativity coefficient (σ) was calculated as the ratio between the two dissociation constants:

$$\sigma =\frac{K_{d,Apo}}{K_{d,inhibitor}},$$

where *K*_*d,Apo*_ is the dissociation constant of PKI(5-24) to apo PKA-C and *K*_*d,inhibitor*_ is the dissociation constant of PKI_5-24_ to the BLU0588-bound enzyme, respectively.

### Determination of Apparent K_i_.

Protein kinase activity was measured with a coupled spectrophotometric kinase assay as described before (20). Measurements were performed with 384-well transparent plates (BRAND, #781680) in a Microplate reader (CLARIOstar Omega; BMG LABTECH) at 340 nm for 580 s. For the apparent K_i_ determination of BLU0588 the reaction mixture of 100 µL contained 100 mM MOPS (pH 7.0), 10 mM MgCl_2_, 1 mM Phosphoenolpyruvate, 15 U/mL Lactate dehydrogenase and 8.4 U/mL Pyruvate kinase (both from rabbit muscle, Roche #10127876001 and #10128163001, respectively), 5 mM 2-Mercaptoethanol, 0.2 mM NADH, 0.2 mg/mL BSA, 1 %DMSO, 20 nM FSS tagged PKA-C and 260 µM of the synthetic peptide Kemptide (Leu-Arg-Arg-Ala-Ser-Leu-Gly, GeneCust) as a substrate. The BLU0588 (MedChemExpress) concentration was varied in the range between 6.4 µM and 12.5 nM employing different ATP concentrations (50 µM to 5 mM) and measured at least in duplicates. Specific activity of the PKA-C was determined via the resulting slope of absorbance per minute. The data were analyzed using a global fit analysis based on the Morrison equation (21, 52, 53) for tight binding inhibitors employing a shared apparent K_i_ value for all ATP datasets (GraphPad Prism v.8.0.1 for Windows, GraphPad Software, Inc.) This equation accounts for the characterization of tight-binding enzyme inhibition because it does not assume that the free concentration of the inhibitor equals the total inhibitor concentration:

$$Y=\nu_{0}\cdot \left(1-\frac{\left[E_{0}\right]+\left[X\right]+\left(K_{i}\cdot \left(1+\left(\frac{S}{K_{m}}\right)\right)\right)-\sqrt{{\left(\left[E_{0}\right]+\left[X\right]+K_{i}\cdot \left(1+\left(\frac{S}{K_{m}}\right)\right)\right)}^{2}-4\left[E_{0}\right]\left[X\right]}}{2\left[E_{0}\right]}\right),$$

where Y is the specific activity of PKA-C calculated from the measured initial rate in the presence of inhibitor, X the corresponding concentration of BLU0588, *v*_0_ the specific activity of PKA-C calculated from the initial rate in the absence of inhibitor, E_0_ is the PKA-C concentration [20 nM], S the respective ATP concentration and K_M_ the Michaelis–Menten constant for ATP for this reaction [15 µM].

### SPR.

The binding of BLU0588 and H89, respectively, to FSS-tagged hCα-subunit was investigated using a Biacore™ 1S+ instrument (Cytiva, Marlborough, MA). A Biacore series S CM5-Chip (Cytiva) was precoated at 25 °C with Strep-Tactin XT (50 µg/mL) to a level of about 2,500 response units (RU) employing the Twin-Strep-tag Capture Kit from IBA Lifesciences GmbH (Göttingen, Germany), following the protocol outlined in the user manual. Subsequently, FSS-hCα was reversibly captured as a ligand on the Strep-Tactin XT sensor surface.

All SPR measurements were performed in 20 mM TRIS (pH 7.4), 150 mM NaCl, 50 µM EDTA, 0.05% Tween20 (Sigma-Aldrich) and 1% DMSO (Carl Roth, Karlsruhe, Germany) at 25 °C. A 7-point solvent correction (0.7 to 1.3% DMSO) was performed as described in the Biacore application guide (Cytiva). At the beginning of each measurement cycle FSS-hCα was captured on one flow cell up to a level of 100 response units (RU) for BLU 0588 and 300 RU for H89, respectively. A flow cell without captured FSS-hCα served as in-line reference for each measurement.

BLU0588 (MedChemExpress, CAS No.: 2810474-78-3) and H89 (MedChemExpress, CAS No.: 130964-39-5) were dissolved in DMSO and stored as a 10 mM stock solution at −80 °C until use.

Interaction analysis was then started by the injection of increasing concentrations of BLU0588 [0.12 to 10 nM, serial threefold dilutions] and H89 [1.2 to 100 nM, serial threefold dilutions] at a flow rate of 100 µL/min for 60 s (association) at 25 °C. Dissociation was then induced by switching to buffer without inhibitor and was monitored for 300 s (BLU0588) and 100 s (H89), respectively. Blank runs were carried out before and after each concentration series, in which only buffer was injected.

Double referencing was performed by subtracting signals from a blank flow cell and blank runs. After each cycle, the sensorchip was regenerated by injecting 3 M Guanidinium chloride solution, 10 mM NaOH with 1.5 M NaCl followed again by 3 M Guanidinium chloride solution for 60 s at a flow rate of 30 μL/min. The determination of the respective rate constants (k_ass_ and k_diss_) employing a 1:1 Langmuir binding model and calculating the experimental R_max_ values was performed with the Biacore Insight (Cytiva) software and sensorgrams were plotted with GraphPad Prism 8.0.1 (GraphPad Software, Inc).

The theoretical binding capacity of the coupled FSS-hCα (theoretical R_max_) was calculated following the equation:

$$\begin{array}{ll} \text{theoretical}\;{\text{R}}_{\text{max}}= & \frac{\text{Molecular}\;{\text{weight}}_{\text{inhibitor}}}{\text{Molecular}\;{\text{weight}}_{\text{FSS}-\text{C}\alpha }}\cdot \\ & {\text{RU}}_{\text{FSS}-\text{C}\alpha }\cdot \text{stoichiometry}\;\text{ratio}, \end{array}$$

$$\begin{array}{ll} \text{theoretical}\;{\text{R}}_{\text{max}}\;\text{for}\;\text{BLU}0588=\; & \frac{423.50\;\text{g}/\text{mol}}{45,200\;\text{g}/\text{mol}}\cdot \\ & 100\;\text{RU}\cdot 1=0.94\;\text{RU}, \end{array}$$

$$\begin{array}{ll} \text{theoretical}\;{\text{R}}_{\text{max}}\;\text{for}\;\text{H}89=\; & \frac{519.28\;\text{g}/\text{mol}}{45,200\;\text{g}/\text{mol}}\cdot \\ & 300\;\text{RU}\cdot 1=3.4\;\text{RU}. \end{array}$$

### IP20 FP Assay.

Binding of the fluorescein-labeled IP20 peptide (FAM-IP20) in the presence and absence of ATP or BLU0588 to PKA-C was assessed with a polarization assay and using buffers as previously described (51). Briefly, FP readings with excitation at 485 nm and emission at 535 nm were carried out with a GENios Pro microplate reader (Tecan) using black flat-bottom Costar® assay plates. Each experiment was tested in triplicate, and the data were analyzed with GraphPad Prism9. To determine the effect of ATP on the binding of FAM-IP20, PKA-C was titrated from 0.25 to 512 nM into assay mix containing 10 mM MgCl_2_, 40 nM FAM-IP20, and ±1 mM ATP. For the comparison of ATP to BLU0588 titration and their effects on FAM-IP20 binding to PKA-C, the FAM-IP20 concentration was at 12 nM, PKA-C at 4 nM, and MgCl_2_ at 10 mM and either ATP or BLU0588 was titrated from 0 to 12.8 µM. We also carried out a BLU0588 titration in the presence ATP (50 µM) added to the assay buffer.

## Supplementary Material

Appendix 01 (PDF)

## Data Availability

Coordinates were submitted to the Protein Data Bank (PDB) with accession number 9PC1 (65). All other data are included in the manuscript and/or *SI Appendix*.
