# Supplementary material for: A PKA-selective inhibitor captures an open but more ordered conformation of the PKA catalytic subunit
Source: Proc Natl Acad Sci U S A. 2026 May 7;123(19):e2536312123. doi: 10.1073/pnas.2536312123 (PMC13167742; doi:10.1073/pnas.2536312123)
Supplement: Supplementary file 1 — Appendix 01 (PDF) [file pnas.2536312123.sapp.pdf]

PNAS

Supporting Information for

A PKA-selective inhibitor captures an open but more ordered conformation of the PKA catalytic subunit.

Jessica G.H. Bruystens, Jian Wu, Gerald Tan, Daniela Bertinetti, Hans-Michael Zenn, Bastian Zimmermann, Lisa Chen, Johannes Köckenberger, Federica Massaro, Banumathi Sankaran, Matthew S. Walters, Gianluigi Veglia, Fleur M. Ferguson, Friedrich W. Herberg, Susan S. Taylor

Corresponding Author: Susan S. Taylor
Email: staylor@ucsd.edu

This PDF file includes:

Figures S1 to S9
Tables S1 to S3
SI Reference 1

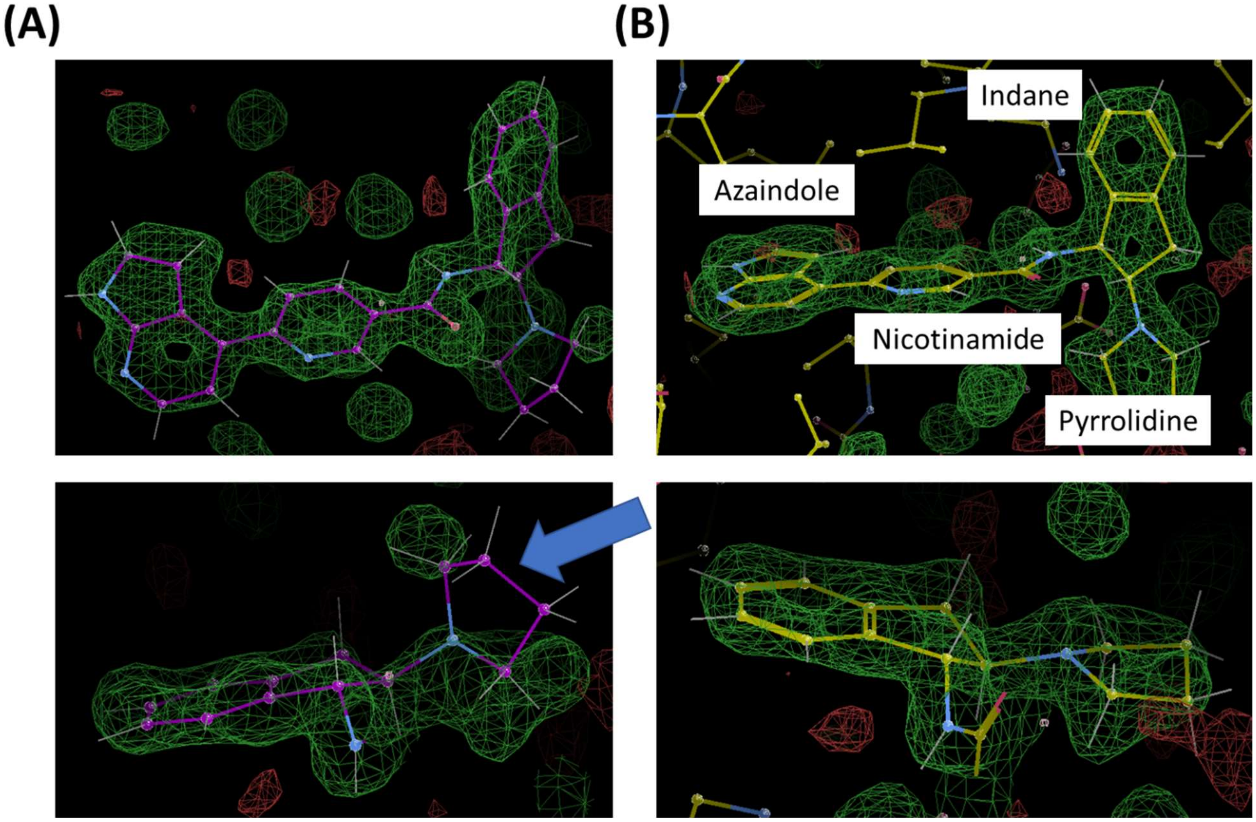

Figure. S1. Fo-Fc omit map at 3s showing the BLU0588 density. (A) BLU0588 predicted puckered geometry (purple) fit into Fo-Fc density (green mesh) shows poor fit of the pyrrolidine ring. **(B)** BLU0588 built into the electron density with coot ligand builder and real space refinement. Density in active site shows BLU0588 geometry with a more planar indane-pyrrolidine moiety (blue arrow). Density images were generated with Coot.

(A)

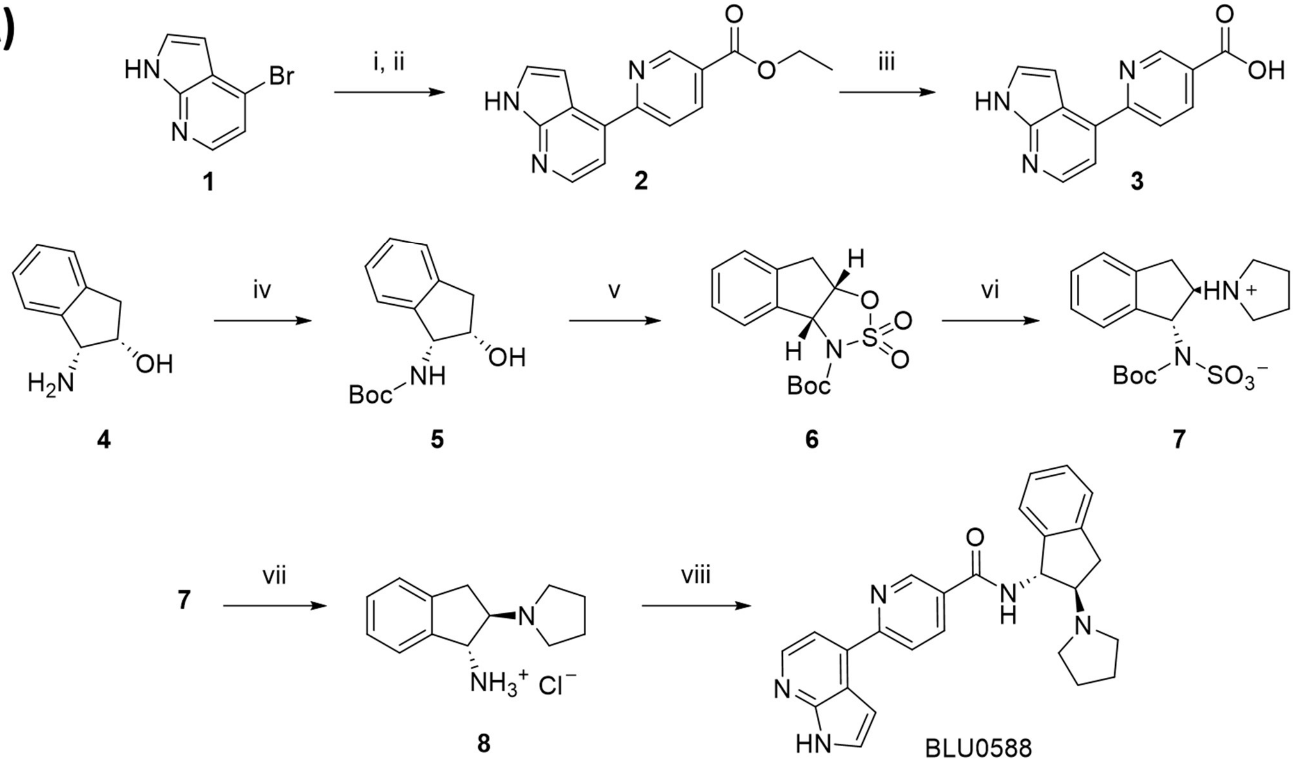

(B)

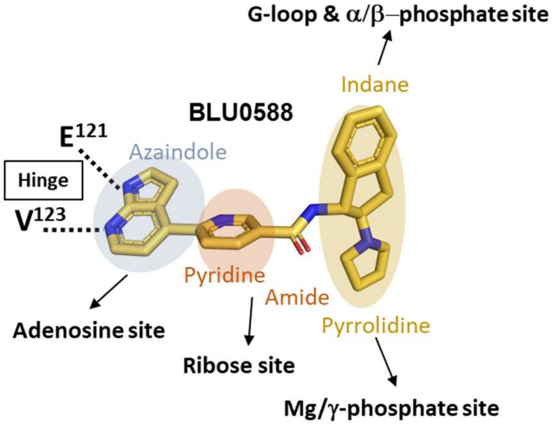

Figure. S2. Synthesis Scheme of BLU0588. (A) The modified synthesis scheme used for the crystallization of BLU0588 is shown and was adapted from the original patented synthesis by Bilfuco et al. (1). i) B_2Pin_2, $Pd(dppf)Cl_2$, KOAc, 1,4-Dioxane, 90 °C, 16 h. ii) ethyl 6-bromonicotinate, $Pd(Amphos)_2Cl_2$,KOAc, 90 °C, 16 h, 38%. iii) NaOH, rt, 3 h, 94%. iv) Boc_2O, TEA, 0 - 25 °C, 16 h, 99%. v) $SOCl_2$, pyridine, -40 °C, 3 h, then: $RuCl_3$ x H_2O, $NaIO_4$, 0°C, 3 h, 39%. vi) pyrrolidine, rt, 16 h, 44%. vii) HCl, rt, 3 h. viii) **3**, HATU, DIPEA, rt, 16 h, 20%. **(B)** Structure and PKA-C binding-surrogate moieties of the BLU0588 inhibitor.

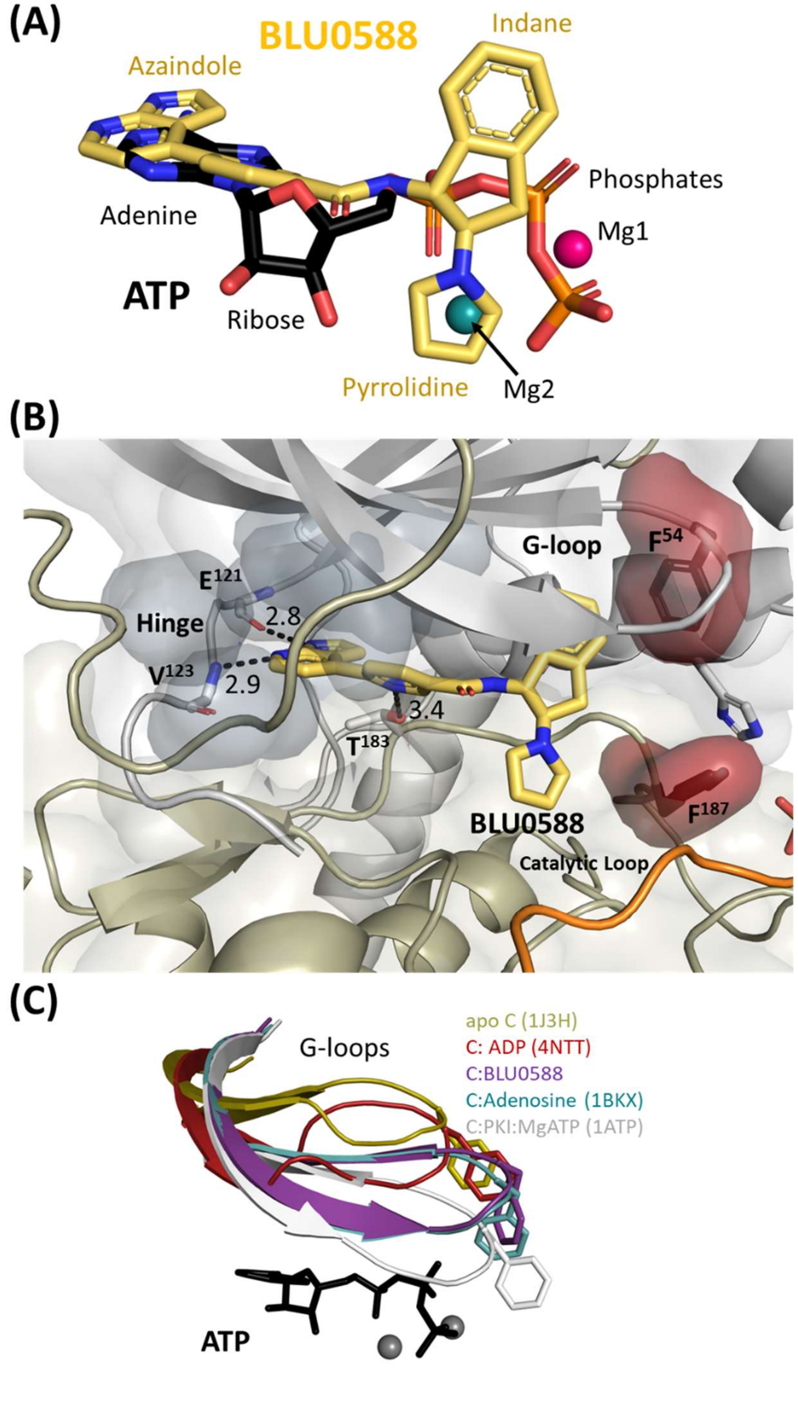

Figure. S3. BLU0588 is bound in the PKA-C active site. (A) 3-D overlay of ATP/Mg^{2+}1 (pink) /Mg^{2+}2 (turquoise) and BLU0588. **(B)** BLU0588 hydrogen bond interactions to the hinge in the adenine pocket and Thr183 of PKA-C. **(C)** BLU0588 induces an upward shift of the G-loop (purple) into a similar position to when adenosine is bound (teal). Position of ATP and magnesiums relative to the G-loops.

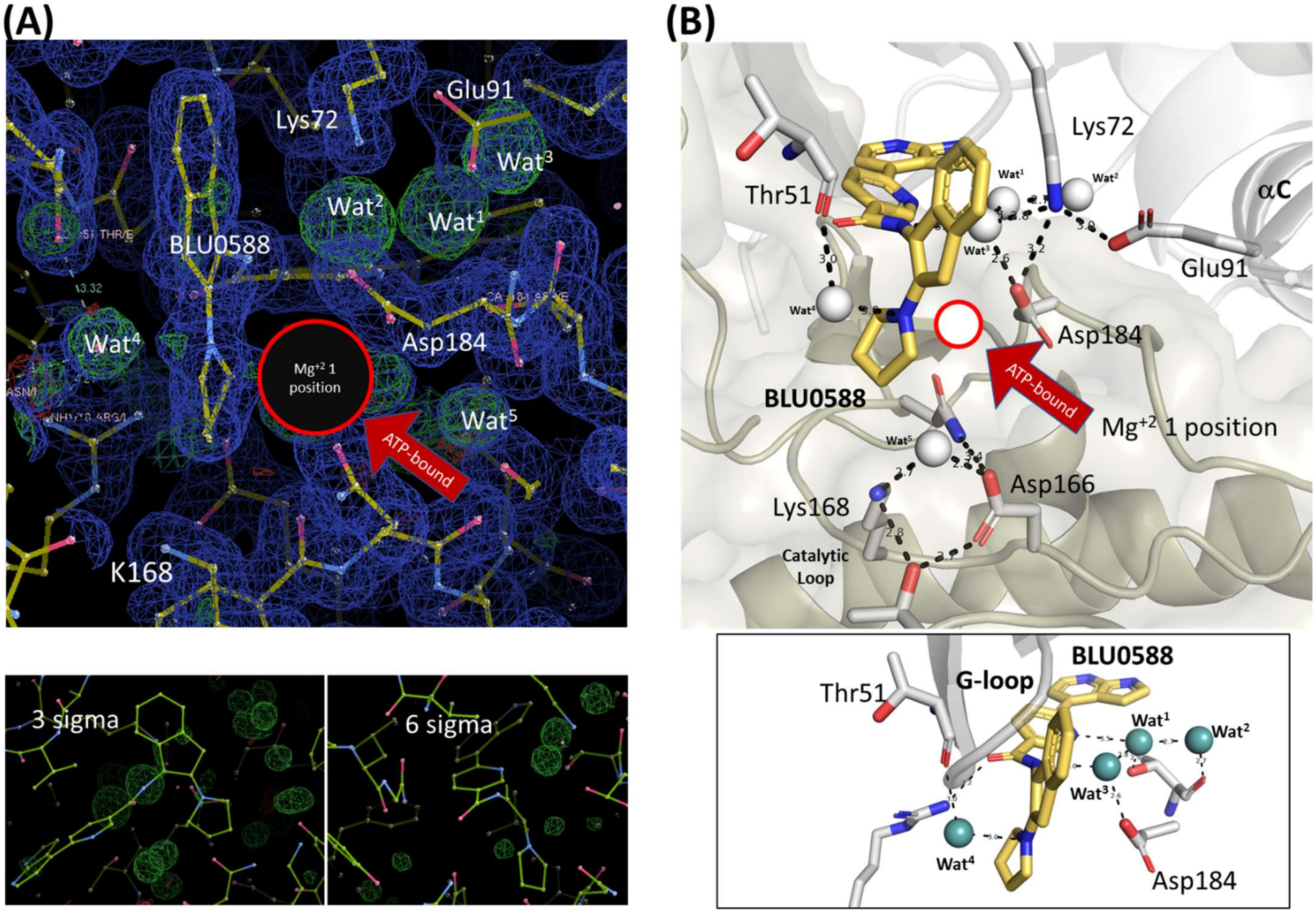

Figure. S4. Electron density of the BLU0588-bound PKA-C active site. (A) Visualization of the three active site water molecules Fo-Fc omit map showing densities at 3s (green mesh) behind Lys72 and Glu91. The corresponding position of metal 1 (Mn in PDB ID: 1ATP) is depicted as a large red circle (top). The active site water molecules are shown at 3 (bottom left) and 6s (bottom right). Density images were generated with Coot. **(B)** Electrostatic contributions in the positioning and coordination of BLU0588 in the metal/phosphate pocket of PKA-C. The linchpin metal space (white disk with red circle) is unoccupied in the BLU0588 bound state.

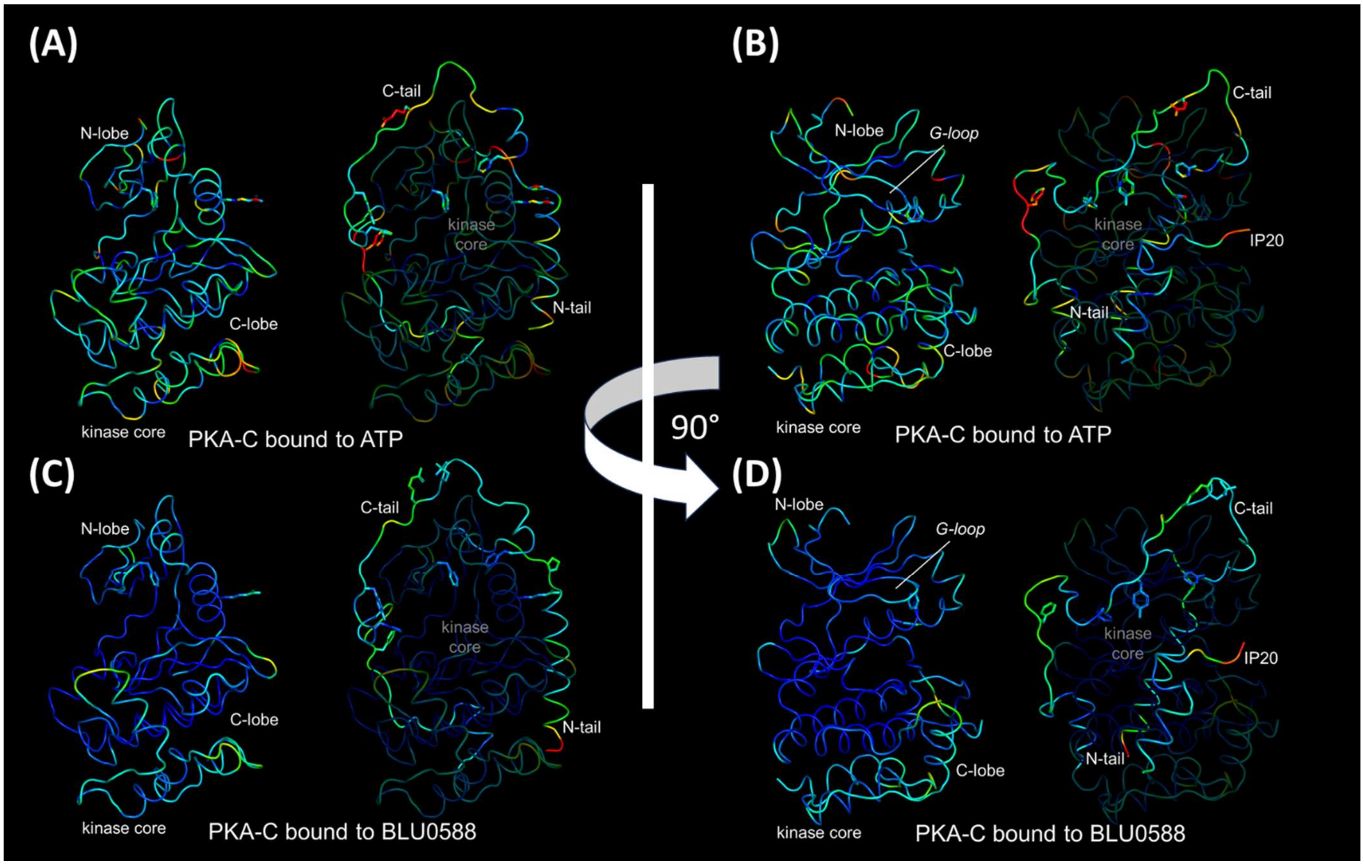

Figure. S5. B-factors for ATP and BLU0588 complexes. The temperature factors show how the extended hydrophobic architecture of both the N- and C-lobes is stabilized by BLU0588: bottom row (**C** and **D**) in comparison to ATP-bound: top row (**A** & **B**). The left panel shows how the N- and C-tails wrap around the kinase core. The right panel shows the standard view of the kinase. In both panels the kinase core is on the left and the tails that flank both lobes of the kinase core are highlighted on the right.

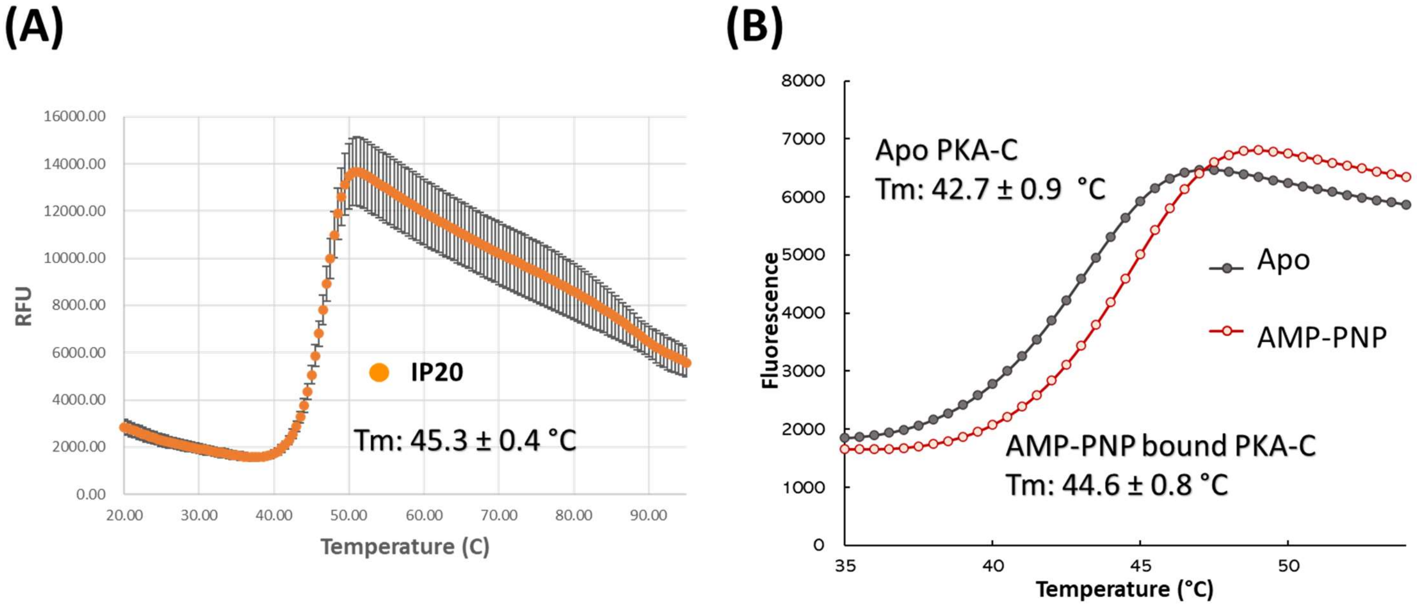

Figure. S6. Thermal shift assays of PKA-C complexes. (A) PKA-C thermal shift assay with IP20. The molar ratio is 1:5 PKA-C:IP20. **(B)** Thermal shift assays of apo and AMP-PNP-bound PKA-C. The molar ratio of protein:nucleotide is 1:50. **(C)** Thermal shift assays of PKA-C bound to H89. The molar ratio is 1:5 enzyme:inhibitor.

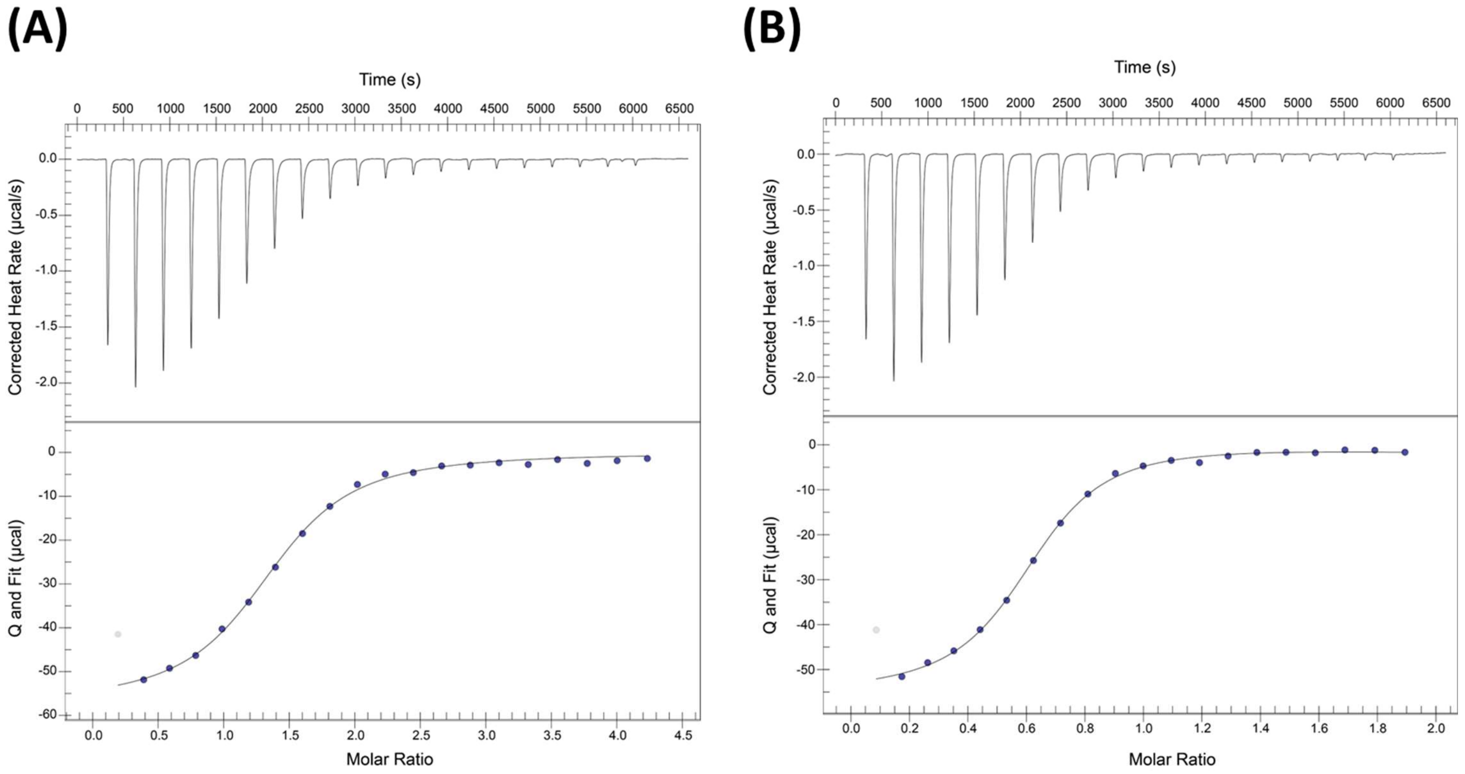

Figure. S7. Isothermal Titration Calorimetry of IP20 to apo compared to BLU0588 saturated PKA-C. (A) Representative ITC binding data for the titration of IP20 into apo PKA-C. **(B)** Representative ITC binding data for the titration of IP20 into BLU0588-bound PKA-C.

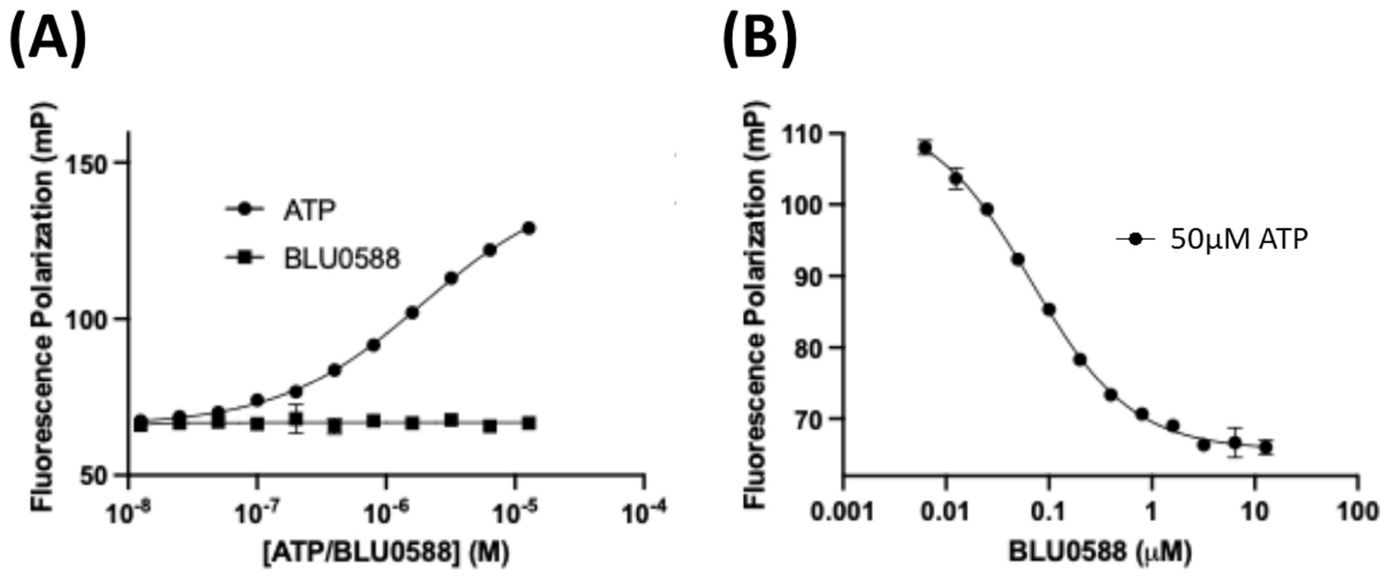

Figure. S8. Fluorescence polarization of IP20 with BLU0588. Fluorescence polarization (FP) assay to analyze binding of fluorescein-labeled IP20 peptide (FAM-IP20) (12nM) to PKA-C (4nM) in 10mM $MgCl_2$. **(A)** FP-assay of PKA-C titrated with 0-12.8μM BLU0588 (black square) or ATP (black circle). **(B)** FP-assay of PKA-C titrated with BLU0588 (0-10μM) in the presence of 50μM ATP. Each experiment was tested in triplicate and the data was analyzed with GraphPad Prism9.

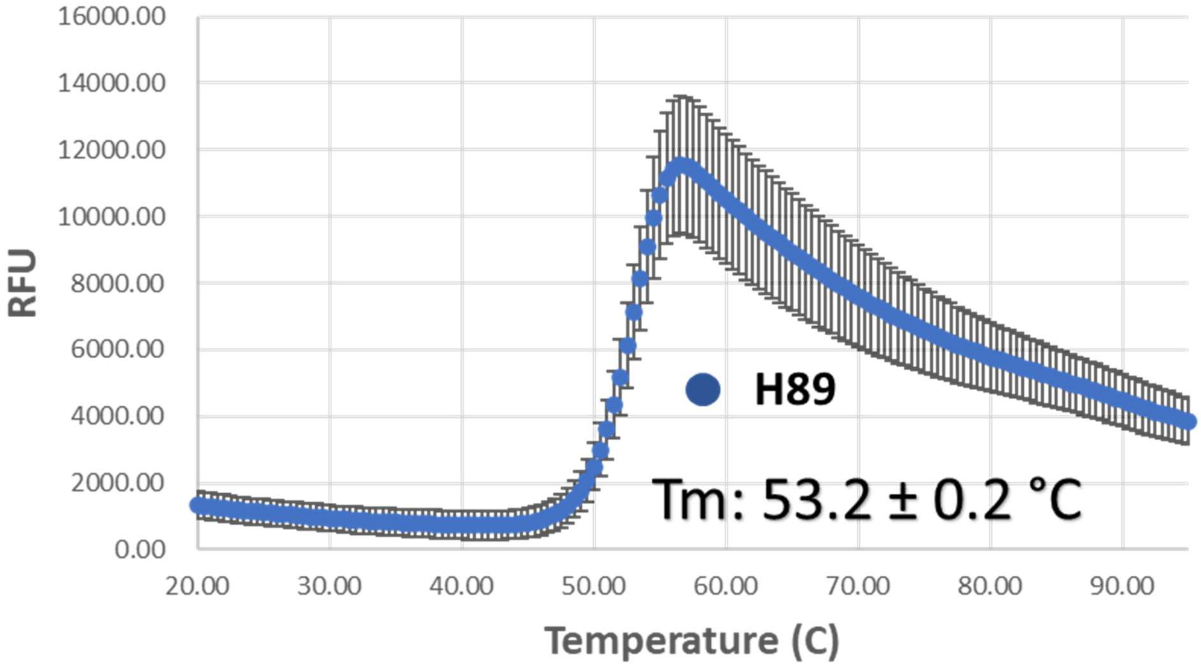

Figure. S9. Thermal shift assay of PKA-C bound to H89. PKA-C thermal shift assay with H89. The molar ration is 1:5 enzyme:inhibitor.

Table S1. Summary of the T_m values obtained from thermal shift assays.

	Tm (°C)
Apo PKA-C	42.7 ± 0.9
PKA-C/AMP-PNP	44.6 ± 0.8
PKA-C/PKI(5-24)	45.3 ± 0.4
PKA-C/H89	53.2 ± 0.2
PKA-C/BLU0588	56.4 ± 0.6
PKA-C/BLU058/PKI(5-24)	57.0 ± 0.7

Table S2. Summary of the dissociation constants and thermodynamics parameters obtained from isothermal titration calorimetry.

PKI(5-24) titration into apo PKA-C			
Kd (μM)	ΔH (kcal/mol)	ΔG (kcal/mol)	TΔS(kcal/mol)
3.8±0.1	-16.8±0.1	-6.83±0.01	-9.91±0.07

PKI(5-24) titration into apo PKA-C bound to BLU0588			
Kd (μM)	ΔH (kcal/mol)	ΔG (kcal/mol)	TΔS(kcal/mol)
2.9±0.1	-15.8±0.4	-7.6±0.1	-8.2±0.5

Cooperativity coefficient

$$\sigma = \frac{K_d^{apo}}{K_d^{BLU0588}} \sim 1.3$$

Table S3. Data Collection and Refinement Statistics. Numbers in parentheses correspond to the highest resolution shell.

**Data collection**	
ALS beamline	8.2.2
Space group	P 21 21 21
Unit cell (a, b, c)	69.515Å 73.132Å 76.535Å
Average Redundancy	11.5 (2.3)
No. of unique reflections	54088 (1578)
Resolution (Å)	42.08-1.55 (1.58-1.55)
Completeness (%)	94.9 (56)
I/sigma	13.8 (1.7)
**Refinement**	
Resolution (Å)	42.08-1.55
R_{work}, R_{free}	0.184, 0.214
Total No. of atoms	3304
Solvent	317
R.m.s. deviations	
Bond lengths (Å)	0.008
Bond angles (º)	0.92
Average B-factor	23.51
**Ramachandran angles (%)**	
most favored (%)	97.7
disallowed	none

SI References

1. Bifulco N, Joseph K, Schalm S, Wilson K, Palmer M, inventorsINHIBITORS OF PROTEIN KINASE A. World Intellectual Property Organization (WIPO) patent WO 2022/165402 A1. 2022.
